# Regression based quasi-experimental approach when randomisation is not an option: interrupted time series analysis

**DOI:** 10.1136/bmj.h2750

**Published:** 2015-06-09

**Authors:** Evangelos Kontopantelis, Tim Doran, David A Springate, Iain Buchan, David Reeves

**Affiliations:** 1 Centre for Health Informatics, Institute of Population Health, University of Manchester, Manchester M13 9GB, UK; 2 NIHR School for Primary Care Research, Centre for Primary Care, Institute of Population Health, University of Manchester, UK; 3 Department of Health Sciences, University of York, UK; 4 Centre for Biostatistics, Institute of Population Health, University of Manchester

## Abstract

Interrupted time series analysis is a quasi-experimental design that can evaluate an intervention effect, using longitudinal data. The advantages, disadvantages, and underlying assumptions of various modelling approaches are discussed using published examples

**Summary points**

Interrupted time series analysis is arguably the “next best” approach for dealing with interventions when randomisation is not possible or clinical trial data are not available
Although several assumptions need to be satisfied first, this quasi-experimental design can be useful in providing answers about population level interventions and effects
However, their implementation can be challenging, particularly for non-statisticians

## Introduction

Randomised controlled trials (RCTs) are considered the ideal approach for assessing the effectiveness of interventions. However, not all interventions can be assessed with an RCT, whereas for many interventions trials can be prohibitively expensive. In addition, even well designed RCTs can be susceptible to systematic errors leading to biased estimates, particularly when generalising results to “real world” settings. For example, the external validity of clinical trials in diabetes seems to be poor; the proportion of the Scottish population that met eligibility criteria for seven major clinical trials ranged from 3.5% to 50.7%.^1^ One of the greatest concerns is patients with multimorbidity, who are commonly excluded from RCTs.^2^

Observational studies can address some of these shortcomings, but the lack of researcher control over confounding variables and the difficulty in establishing causation mean that conclusions from studies using observational approaches are generally considered to be weaker. However, with quasi-experimental study designs researchers are able to estimate causal effects using observational approaches. Interrupted time series (ITS) analysis is a useful quasi-experimental design with which to evaluate the longitudinal effects of interventions, through regression modelling.^3^ The term quasi-experimental refers to an absence of randomisation, and ITS analysis is principally a tool for analysing observational data where full randomisation, or a case-control design, is not affordable or possible. Its main advantage over alternative approaches is that it can make full use of the longitudinal nature of the data and account for pre-intervention trends (fig 1). This design is particularly useful when “natural experiments” in real word settings occur—for example, when a health policy change comes into effect. However, it is not appropriate when trends are not (or cannot be transformed to be) linear, the intervention is introduced gradually or at more than one time point, there are external time varying effects or autocorrelation (for example, seasonality), or the characteristics of the population change over time—although all these can be potentially dealt with through modelling if the relevant information is known.

**Figure fig1:**
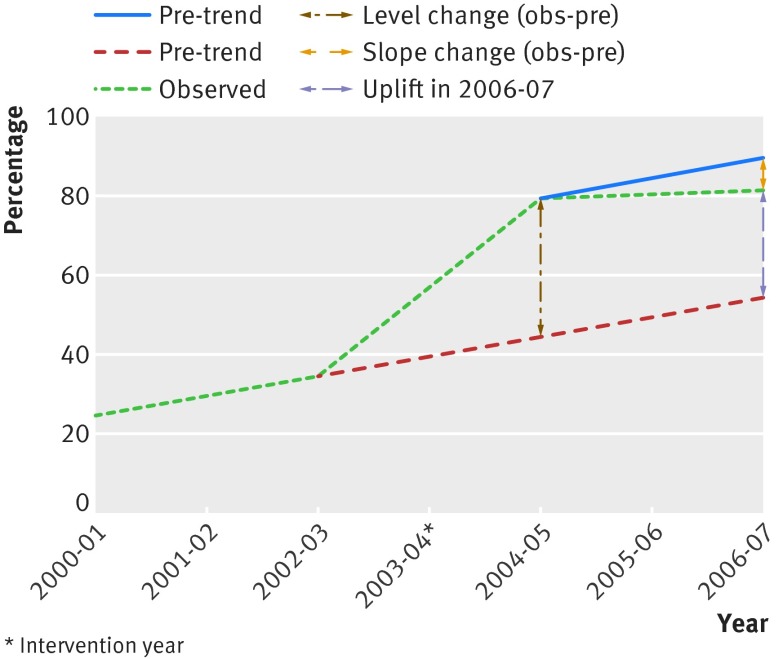
**Fig 1** Interrupted time series analysis components in relation to the Quality and Outcomes Framework intervention

Variations on this design are also known as segmented regression or regression discontinuity analysis and have been described elsewhere,^4^ but we will focus on longitudinal data and practical modelling. ITS encompasses a wide range of modelling approaches and we describe the steps required to perform simple or more advanced analyses, using previously published analyses from our research group as examples.

## The question

We demonstrate a range of ITS models using the “natural experiment” of the introduction of the Quality and Outcomes Framework (QOF) pay for performance scheme in UK primary care. The QOF was introduced in the 2004-05 financial year by the UK government to reward general practices for achieving clinical targets across a range of chronic conditions, as well as other more generic non-clinical targets. This large scale intervention was introduced nationally, without previous assessment in an experimental setting. Because of the great financial rewards it offered, it was adopted almost universally by general practitioners, despite its voluntary nature.

A fundamental research question concerned the effect of this national intervention on quality of care, as measured by the evidence based clinical indicators included in the incentivisation scheme. In operational form, did performance on the incentivised activities improve by the third year of the scheme (2006-07), compared with two years before its introduction (2002-03)? For our analyses we considered the year immediately before the scheme’s introduction (2003-04) to be a preparatory year, as information about the proposed targets was available to practices and this might have affected performance. A basic pre-post analysis would involve an unadjusted or adjusted comparison of mean levels of quality of care across the two comparator years—for example, with a *t* test or a linear regression controlling for covariates. However, such analyses would fail to account for any trends in performance before the intervention—that is, changes in levels of care from 2000-01 to 2002-03. Importantly, in the context of the QOF, previous performance trends cannot be assumed to be negligible, since quality standards for certain chronic conditions included in the scheme (for example, diabetes) were published in 2001 or earlier. This is where the strength of the ITS approach lies; to evaluate the effect of the intervention accounting for the all important pre-intervention trends (table[Table tbl1]).

**Table tbl1:** Introduction of the Quality and Outcomes Framework, summary of examples

Question	Assumptions and limitations*	Approach	Findings	Difficulty
What was the effect of the intervention on quality of care for asthma, diabetes, and heart disease?	Linear trends; no account for population changes over time; no control group	Regression modelling with the three ITS components: pre-intervention slope, level change and change in slope (web appendix 1)	The intervention had an effect on quality of care for diabetes and asthma but not for heart disease, by the third year of the scheme (2006-07)^5^	Simple
Did the intervention effect on diabetes vary by patient characteristics?	Linear trends; no account for population changes over time; no control group	Advanced: regression modelling with the three ITS components, interacted with each of the patient characteristics (web appendix 2)	The effect of the intervention varied for number of years living with the condition with the smallest gains observed for newly diagnosed cases, but not for age, gender, or comorbidities^8^	Advanced
Did the intervention lead to deterioration in non-incentivised aspects of care?	Linear trends; no control group	Expert: multistage regression modelling (web appendix 3)	Improvements attributed to financial incentives appeared to have been achieved at the expense of small detrimental effects on non-incentivised aspects of care^9^	Expert
Did the removal of the incentive lead to deterioration in previously incentivised aspects of care?	Linear trends	Expert: multistage regression modelling and meta-analysis (web appendix 4)	The partial withdrawal of incentives had little or no effect on quality of recorded care^10^	Expert

ITS=interrupted time series.

*In addition to potential external time varying effects or autocorrelation.

## Approaches

We describe the processes, assumptions, and limitations across four ITS modelling approaches, starting with the simplest and concluding with the most complex. Code scripts in Stata are provided for all examples (web appendices 1-4).

### Basic

In its simplest form, an ITS is modelled using a regression model (such as linear, logistic, or Poisson) that includes only three time based covariates, whose regression coefficients estimate the pre-intervention slope, the change in level at the intervention point, and the change in slope from pre-intervention to post-intervention. The pre-intervention slope quantifies the trend for the outcome before the intervention. The level change is an estimate of the change in level that can be attributed to the intervention, between the time points immediately before and immediately after the intervention, and accounting for the pre-intervention trend. The change in slope quantifies the difference between the pre-intervention and post-intervention slopes (fig 1). The key assumption we have to make is that without the intervention we set out to quantify, the pre-intervention trend would continue unchanged into the post-intervention period and there are no external factors systematically affecting the trends (that is, other “interventions”).

We collected performance data on asthma, diabetes, and coronary heart disease from 42 general practices for four time points: 1998 and 2003 (pre-intervention) and 2005 and 2007 (post-intervention). This was the setup for the 2009 analysis of the Quality in Practice (QuIP) study.^5^ We generated the three ITS specific variables and used linear regression modelling. The analysis allowed us to quantify the effect of the intervention on recorded quality of care in the three conditions of interest, on top of what would be expected from the observed pre-intervention trend. We found that the intervention had an effect on quality of care for diabetes and asthma but not for heart disease (fig 2A[Fig fig2]). Since observations over time within each general practice can be treated as correlated, we used a multilevel regression model to account for clustering of observations within practices.^6^ Bootstrap techniques can also be used to obtain more robust standard errors for the estimates.^7^

**Figure fig2:**
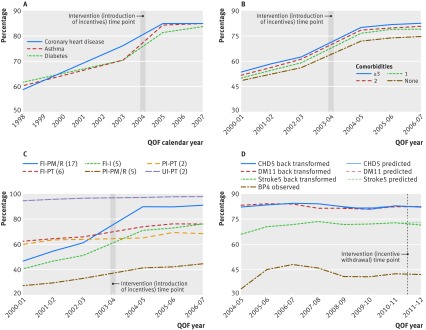
**Fig 2** Quality and Outcomes Framework (QOF) performance graphs for four presented examples. (A) Care for asthma, diabetes, and heart disease. Aggregate practice level performance across three clinical domains of interest.^5^ (B) Diabetes care by number of comorbidities. Aggregate patient level performance for patients in the diabetes domain, by number of additional conditions.^8^ (C) Incentivised and non-incentivised aspects of care. Aggregate practice level performance by incentivisation category and indicator type.^9^ (D) Blood pressure measurement indicators. Aggregate practice level performance on blood pressure measurement indicator.^10^ FI=fully incentivised, PI=partially incentivised, UI=unincentivised, PM/R=process measurement recording, PT=process treatment, I=intermediate outcome. The number of indicators in each group are in parentheses. CHD, DM, Stroke, and BP relate to the coronary heart disease, diabetes mellitus, stroke, and hypertension QOF clinical domains, respectively

Three important assumptions accompany this form of ITS analysis. Firstly, pre-intervention trends are assumed to be linear. Linearity of trends over time needs to be evaluated and confirmed firstly through visualisation and secondly with appropriate statistical tools for the ITS analysis results to have any credence. However, validating linearity can be a problem when there are only a few pre-intervention time points and is impossible with only two. Secondly, the ITS model estimates have not been controlled for covariates. The models assume that the characteristics of the populations remain unchanged throughout the study period and changes in the population base that might explain changes in the outcome are not accounted for. Thirdly, there is no comparator against which to adjust the results for changes that should not be attributed to the intervention itself.

### Advanced

With some modelling changes one can evaluate whether the intervention varies in relation to population characteristics (practices or patients, in the QOF context). For example, we can assess whether the impact of the QOF on performance of incentivised activities (HbA_1c_ control ≤7.4% or HbA_1c_ control ≤10% and retinal screening for patients with diabetes) varies by age group or other patient or practice characteristics.^8^ To accomplish this we included “interaction terms” between the covariate (characteristics) of interest and the three ITS components relating to the pre-intervention slope, level change, and change in slope. A separate model needs to be executed for each covariate of interest.

In addition, the estimated pre-intervention slope can be used to compute predictions of what the value of the outcome would have been at post-intervention time points if the intervention had not taken place. These estimates can then be compared against observations for a specific time point, and an overall difference, or “uplift” (fig 1), attributed to the intervention obtained. This comparison between predictions and observations not only applies to the advanced models, where both main and interaction effects estimates need to be considered, but to simple models as well. Using this approach we found that composite quality for patients with diabetes improved over and above the pre-incentive trend in the first post-intervention year, but by the third year improvement was smaller. The effect of the intervention did not vary by age, sex, or multimorbidity (fig 2B[Fig fig2]) but did for number of years living with the condition, with the smallest gains observed for newly diagnosed cases.^8^ However, the linearity assumption, the lack of adjustment for changes in the population characteristics over time, and the absence of a comparator still apply.

### Expert

More flexible modelling options are possible in which we can overcome some of the limitations in the basic and advanced designs. Let us assume a patient level analysis of incentivised and non-incentivised aspects of quality of care across a range of clinical indicators, with our aim being to evaluate whether the effect of the QOF on performance varies across fully incentivised and non-incentivised indicators.^9^ Using regression modelling we can evaluate the relations between the outcome and covariates of interest (for example, patient age and sex), to obtain estimates that are adjusted for population changes, at specific time points. For example, to calculate the adjusted increase in the outcome above the projected trend, in the first post-intervention year. However, the modelling complexities are formidable and involve numerous steps. Using this approach we found that improvements attributed to financial incentives were achieved at the expense of small detrimental effects on non-incentivised aspects of care (fig 2C[Fig fig2]).^9^

An alternative modelling approach can additionally incorporate “control” factors into the analyses. Let us assume we want to investigate the effect of withdrawing a financial incentive on practice performance.^10^ In 2012-13, the QOF underwent a major revision and six clinical indicators were removed from the incentivisation scheme: blood pressure monitoring for coronary heart disease, diabetes, and stroke; cholesterol concentration monitoring for coronary heart disease and diabetes; blood glucose monitoring for diabetes. We used a regression based ITS to quantify the effect of the intervention, in this case the withdrawal of the incentive. We grouped the indicators by process and analysed these as separate groups, including indicators with similar characteristics that remained in the scheme and could act as “controls.” A multilevel mixed effects regression was used to model performance on all these indicators over time, controlled for covariates of interest and including an interaction term between time and indicators, but excluding post-intervention observations for the withdrawn indicators. Predictions and their standard errors were then obtained from the model, for the withdrawn indicators post-intervention and for each practice. These were compared with actual post-intervention observations using advanced meta-analysis methods,^11^ to account for variability in the predictions, and obtain estimates of the differences. We found that the withdrawal of the incentive had little or no effect on quality of recorded care (fig 2D[Fig fig2]).^10^

## Discussion

Although randomised controlled trials (RCTs) are considered the ideal approach for assessing the effectiveness of many interventions, we argue that observational data still need to be harnessed and utilised though robust alternative designs, even where trial evidence exists. Large scale population studies, using primary care databases, for example, can be valuable complements to well designed RCT evidence.^12^ Sometimes evaluation through randomisation is not possible at all, as was the case with the UK’s primary care pay for performance scheme, which was implemented simultaneously across all UK practices. In either case, well designed observational studies can contribute greatly to the knowledge base, albeit with careful attention required to assess potential confounding and other threats to validity.

To better describe the methods, we drew on examples from our QOF research experiences. This approach allowed us to describe designs of increasing complexity, as well as present their technical details in the appendix code. However, we should also clarify that the ITS design is much more than a tool for QOF analyses, and it can investigate the effect of any policy change or intervention in a longitudinal dataset, provided the underlying assumptions are met. For example, it can investigate the decline in pneumonia admissions after routine childhood immunisation with pneumococcal conjugate vaccine in the United States,^13^ the effect of 20 mph traffic zones on road injuries in London,^14^ or the impact of infection control interventions and antibiotic use on hospital meticillin resistant *Staphylococcus aureus* (MRSA) in Scotland.^15^

Quasi-experimental designs, and ITS analyses in particular, can help us unlock the potential of “real world” data, the volume and availability of which is increasing at an unprecedented rate. The limitations of quasi-experimental studies are generally well understood by the scientific community, whereas the same might not be true of the shortcomings of RCTs. Although the limitations can be daunting, including autocorrelation, time varying external effects, non-linearity, and unmeasured confounding, quasi-experimental designs are much cheaper and have the capacity, when carefully conducted, to complement trial evidence or even to map uncharted territory.

**Sources and selection criteria**

We chose to present examples that we ourselves have presented in major clinical journals, including *The BMJ*
EK and DR are experienced statisticians and health services researchers who have published numerous clinical papers using the described methods. TD is a professor of public health with considerable experience in these methods, who has co-authored most of these publications. DAS is research fellow in health informatics, a more recent addition to the research group, who co-authored our latest interrupted time series analysis. IB is professor of health informatics with wide experience in statistical methodology and its practical implementation

## Notes

Provenance and peer review: Not commissioned; externally peer reviewed.

## Notes

Cite this as: *BMJ* 2015;350:h2750
