# Supplementary material for: Regression based quasi-experimental approach when randomisation is not an option: interrupted time series analysis
Source: BMJ. 2015 Jun 9;350:h2750. doi: 10.1136/bmj.h2750 (PMC4460815; doi:10.1136/bmj.h2750)
Supplement: Supplementary file 1 [file kone024026.ww1_default.pdf]

Web appendix 1: Code file for simple ITS analysis

```
/*program that runs interrupted time series (segmented) linear regression
- problem is that effects are fixed*/
program ITSfixed
    local txt1 = "P:\Evan\Quip\2008analysis_4timepoints\main\"
    /*first argument: the name of the variable (angscore or angnsf)*/
    if "`1'"=="" {
        display "you need to include the dependent variable's name as the
first argument"
        error 197
    }
    /*second argument optional. if "boot" is inputted, bootstrapped
regressions are executed instead*/
    local bopt = 0
    if "`2'"=="boot" {
        local bopt=1
    }
    /*caclulate substring needed*/
    local ss1 = substr("`1'",1,3)
    /*load dataset and keep what we need*/
    use `txt1'\`ss1'_pract.dta, clear
    keep pract_id `1'*
    /*rename variables appropriately so that reshaping can take place*/
    foreach x in 0 5 7 9 {
        local yr = 1998 + `x'
        /*original percentages*/
        rename `1'_`x' `1'`yr'
        /*transformed scores*/
        rename `1'_`x'L `1'L`yr'
    }
    /*reshape to the form needed by the regression*/
    qui reshape long `1' `1'L, i(pract_id) j(year)
    qui keep pract_id `1' `1'L year
    /*make 2004 year 0*/
    qui gen cyear = year-2004
    /*dummy variable - binary intervention*/
    qui gen level_change = 0
    qui replace level_change = 1 if year>2004
    /*dummy variable - interaction term - start counting AFTER 1st
intervention or post-intervention point*/
    /*after 1st time point where the intervention has an effect*/
    qui gen slope_change = level_change*(year-2005)
    label var slope_change "Change in slope, post-contract"
    /*rename variables for better output*/
    qui rename cyear slope_pre
    /*"plain" regressions*/
    if `bopt'==0 {
        /*regression - on percentages*/
        di _newline(2) "`1' - segmented regression (ITS) on observed
scores (linear)"
        regress `1' level_change slope_pre slope_change, robust
cluster(pract_id)
        /*regression - on transformed scores*/
        di _newline(2) "`1' - segmented regression (ITS) on transformed
scores (logit)"
```

```
        regress `1'L level_change slope_pre slope_change, robust
cluster(pract_id)
    }
    else {
        /*randomly set the seed number*/
        local time = c(current_time) /*get time to add to seed number*/
        local time : subinstr local time ":" "", all /*remove all the
:s*/
        local seednum = 123400 + `time'
        /*regression - on percentages*/
        di _newline(2) "`1' - bootstrapped segmented regression (ITS) on
observed scores - linear, 10000 iterations"
        bootstrap, reps(10000) seed(`seednum') nodots: regress `1'
level_change slope_pre slope_change, robust cluster(pract_id)
        /*regression - on transformed scores*/
        di _newline(2) "`1' - bootstrapped segmented regression (ITS) on
transformed scores - logit, 10000 iterations"
        bootstrap, reps(10000) seed(`seednum') nodots: regress `1'L
level_change slope_pre slope_change, robust cluster(pract_id)
    }
end

/*program that runs interrupted time series (segmented) linear regression
for each practice then saves coefficients*/
/*and compares with t-test*/
program ITScustom
    local txt1 = "P:\Evan\Quip\2008analysis_4timepoints\main\"
    /*first argument: the name of the variable (angscore or angnsf)*/
    if "`1'"=="" {
        display "you need to include the dependent variable's name as the
first argument"
        error 197
    }
    /*second argument optional. if "boot" is inputted, bootstrapped
regressions are executed instead*/
    local bopt = 0
    if "`2'"=="boot" {
        local bopt=1
    }
    /*caclulate substring needed*/
    local ss1 = substr("`1'",1,3)
    /*load dataset and keep what we need*/
    use `txt1'\`ss1'_pract.dta, clear
    keep pract_id `1'*
    /*rename variables appropriately so that reshaping can take place*/
    foreach x in 0 5 7 9 {
        local yr = 1998 + `x'
        /*original percentages*/
        rename `1'_`x' `1'`yr'
        /*transformed scores*/
        rename `1'_`x'L `1'L`yr'
    }
    /*reshape to the form needed by the regression*/
    qui reshape long `1' `1'L, i(pract_id) j(year)
    qui keep pract_id `1' `1'L year
    /*make 2004 year 0*/
    qui gen cyear = year-2004
    /*dummy variable - binary intervention*/
```

```
    qui gen level_change = 0
    qui replace level_change = 1 if year>2004
    /*dummy variable - interaction term - start counting AFTER 1st
intervention or post-intervention point*/
    /*after 1st time point where the intervention has an effect*/
    qui gen slope_change = level_change*(year-2005)
    label var slope_change "Change in slope, post-contract"
    /*rename variables for better output*/
    qui rename cyear slope_pre
    /*loop to go through all practices*/
    local cnt=0
    forvalues i=1(1)60 {
        qui count if pract_id==`i'
        if `r(N)'>0 {
            local cnt = `cnt' + 1
            local pid`cnt' = `i'
            /*"plain" regressions*/
            /*regression - on percentages*/
            qui regress `1' level_change slope_pre slope_change if
pract_id==`i', robust cluster(pract_id)
            /*grab coefficients and put them in scalars*/
            matrix temp = e(b)
            /*level change*/
            local a`cnt' = temp[1,1]
            /*slope pre*/
            local b`cnt' = temp[1,2]
            /*slope change*/
            local c`cnt' = temp[1,3]
            /*_cons*/
            local d`cnt' = temp[1,4]
            /*regression - on transformed scores*/
            qui regress `1'L level_change slope_pre slope_change if
pract_id==`i', robust cluster(pract_id)
            /*grab coefficients and put them in scalars*/
            matrix temp = e(b)
            /*level change*/
            local e`cnt' = temp[1,1]
            /*slope pre*/
            local f`cnt' = temp[1,2]
            /*slope change*/
            local g`cnt' = temp[1,3]
            /*_cons*/
            local h`cnt' = temp[1,4]
        }
    }
    /*create datasets with coefficients*/
    qui drop _all
    qui set obs `cnt'
    qui gen pract_id=.
    qui gen level_change=.
    qui gen slope_pre=.
    qui gen slope_change=.
    qui gen intercept04=.
    qui gen level_changeL=.
    qui gen slope_preL=.
    qui gen slope_changeL=.
    qui gen intercept04L=.
    forvalues i=1(1)`cnt' {
```

```
        qui {
            replace pract_id = `pid`i'' in `i'
            replace level_change = `a`i'' in `i'
            replace slope_pre = `b`i'' in `i'
            replace slope_change = `c`i'' in `i'
            replace intercept04 = `d`i'' in `i'
            replace level_changeL = `e`i'' in `i'
            replace slope_preL = `f`i'' in `i'
            replace slope_changeL = `g`i'' in `i'
            replace intercept04L = `h`i'' in `i'
        }
    }
    if `bopt'==0 {
        /*headers*/
        di _newline(2) "`1' - simple t-test results"
        di _col(20) "p-value" _col(32) "t-value" _col(44) "SE"
        /*simple t-tests*/
        foreach x in level_change slope_pre slope_change level_changeL
slope_preL slope_changeL {
            qui ttest `x'==0
            di "`x'" _col(20) %7.6f r(p) _col(32) %7.6f r(t) _col(44)
%7.6f r(se)
        }
    }
    else {
        /*headers*/
        di _newline(2) "`1' - bootstrapped t-test results"
        di "note: displaying CI and p-value of the bootsrapped mean"
        di _col(20) "mean" _col(32) "95%CI-lo" _col(44) "95%CI-up"
_col(56) "p-value"
        /*bootsrapped t-tests*/
        foreach x in level_change slope_pre slope_change level_changeL
slope_preL slope_changeL {
            qui bootstrap mval=r(mu_1), nodots rep(1000): ttest `x'==0
            scalar zvalue = _b[mval]/_se[mval]
            scalar pvalue = 2*normal(-abs(zvalue))
            matrix temp = e(ci_normal)
            scalar mvallo = temp[1,1]
            scalar mvalup = temp[2,1]
            di "`x'" _col(20) %7.6f _b[mval] _col(32) %7.6f mvallo
_col(44) %7.6f mvalup _col(56) %7.6f pvalue
        }
    }
end

/*program that runs interrupted time series (segmented) random effects*/
program ITSrandom
    local txt1 = "P:\Evan\Quip\2008analysis_4timepoints\main\"
    /*first argument: the name of the variable (angscore or angnsf)*/
    if "`1'"=="" {
        display "you need to include the dependent variable's name as the
first argument"
        error 197
    }
    /*second argument linear or logit*/
    local linlog = "linear"
    if "`2'"=="logit" {
        local linlog = "logit"
```

```
    }
    /*third argument optional. if "boot" is inputted, bootstrapped
regressions are executed instead*/
    local bopt = 0
    if "`3'"=="boot" {
        local bopt=1
    }
    /*caclulate substring needed*/
    local ss1 = substr("`1'",1,3)
    /*load dataset and keep what we need*/
    use `txt1'\`ss1'_pract.dta, clear
    keep pract_id `1'*
    /*rename variables appropriately so that reshaping can take place*/
    foreach x in 0 5 7 9 {
        local yr = 1998 + `x'
        /*original percentages*/
        rename `1'_`x' `1'`yr'
        /*transformed scores*/
        rename `1'_`x'L `1'L`yr'
    }
    /*reshape to the form needed by the regression*/
    qui reshape long `1' `1'L, i(pract_id) j(year)
    qui keep pract_id `1' `1'L year
    /*make 2004 year 0*/
    qui gen cyear = year-2004
    /*dummy variable - binary intervention*/
    qui gen level_change = 0
    qui replace level_change = 1 if year>2004
    /*dummy variable - interaction term - start counting AFTER 1st
intervention or post-intervention point*/
    /*after 1st time point where the intervention has an effect*/
    qui gen slope_change = level_change*(year-2005)
    label var slope_change "Change in slope, post-contract"
    /*rename variables for better output*/
    qui rename cyear slope_pre
    qui count if `1'!=.
    if `r(N)'>=8 {
        /*"plain" regressions*/
        if `bopt'==0 {
            if "`linlog'"=="linear" {
                /*regression - on percentages*/
                di _newline(2) "`1' - segmented RE xtreg regression (ITS)
on observed scores (linear)"
                /*xtmixed `1' level_change slope_pre slope_change ||
pract_id: level_change slope_pre slope_change*/
                xtreg `1' level_change slope_pre slope_change,  re robust
i(pract_id) cluster(pract_id)
            }
            else {
                /*regression - on transformed scores*/
                di _newline(2) "`1' - segmented RE xtreg regression (ITS)
on transformed scores (logit)"
                qui count if `1'L!=.
                xtreg `1'L level_change slope_pre slope_change,  re
robust i(pract_id) cluster(pract_id)
            }
        }
        else {
```

```
            /*randomly set the seed number*/
            local time = c(current_time) /*get time to add to seed
number*/
            local time : subinstr local time ":" "", all /*remove all the
:s*/
            local seednum = 123410 + `time'
            if "`linlog'"=="linear" {
                /*regression - on percentages*/
                di _newline(2) "`1' - bootstrapped RE xtreg segmented
regression (ITS) on observed scores - linear, 1000 iterations"
                bootstrap, reps(1000) seed(`seednum') nodots: xtreg `1'
level_change slope_pre slope_change,  re robust i(pract_id) /*
                */cluster(pract_id)
            }
            else {
                /*regression - on transformed scores*/
                di _newline(2) "`1' - bootstrapped RE xtreg segmented
regression (ITS) on transformed scores - logit, 1000 iterations"
                bootstrap, reps(1000) seed(`seednum') nodots: xtreg `1'L
level_change slope_pre slope_change,  re robust i(pract_id) /*
                */cluster(pract_id)
            }
        }
        /*postestimation - comparison of trans with post slope*/
        /* level_change over two years so to get the trans qof trend
change we divide by 2*/
        qui lincom slope_change - level_change/2
        /*get estimated difference and confidence interval*/
        scalar ldiff_est = r(estimate)
        scalar ldiff_se = r(se)
        scalar ldiff_lo = ldiff_est - 1.96*ldiff_se
        scalar ldiff_up = ldiff_est + 1.96*ldiff_se
        /*get p-value*/
        scalar t = r(estimate)/r(se)
        qui count
        scalar df = r(N)-1
        scalar pval = 2*ttail(df,abs(t))
        /*output*/
        di _newline(2) "`1' - `linlog' model" _col(40) "diff" _col(50)
"lowerCI" _col(60) "upperCI" _col(70) "p"
        di "trans-QOF (03-05) estimate vs obs 07" _col(40) %5.3f
ldiff_est _col(50) %5.3f ldiff_lo _col(60) %5.3f /*
        */ldiff_up _col(70) %5.3f pval
    }
    else {
        di "`1': not enough observations for regression"
    }
end

/*useful for going through all indicators and creating result files*/
program allrun
    set more off
    if "`1'"==""{
        display "you need to include angina asthma or diabetes as first
argument"
        error 197
    }
    if "`1'"!="angina" & "`1'"!="asthma" & "`1'"!="diabetes" {
```

```
        display "you need to include angina asthma or diabetes as first
argument"
        error 197
    }
    /*second argument linear or logit*/
    local linlog = "linear"
    if "`2'"=="logit" {
        local linlog = "logit"
    }
    /*third argument optional. if "boot" is inputted, bootstrapped
regressions are executed instead*/
    local bopt = ""
    if "`3'"=="boot" {
        local bopt="boot"
    }
    /*caclulate substring needed*/
    local ss1 = substr("`1'",1,3)
    local txt1 = "P:\Evan\Quip\2008analysis_4timepoints\main\"
    /*indicator loop*/
    forvalues i=101(1)123 {
        use `txt1'\`ss1'_pract.dta, clear
        /*verify that methods will only be executed for variables that
are there*/
        capture confirm variable `ss1'`i'_9
        if _rc==0 {
            ITSrandom `ss1'`i' `linlog' `bopt'
        }
    }
    foreach x in "inc" "ninc" {
        qui use `txt1'\`ss1'_pract.dta, clear
        ITSrandom `ss1'`x' `linlog' `bopt'
    }
    if "`ss1'" == "ast" {
        ITSrandom `ss1'score `linlog' `bopt'
    }
    else {
        ITSrandom `ss1'nsf `linlog' `bopt'
    }
end

/*backtrasform logit differences to percentages including confidence
intervals*/
program backt
    if "`1'"==""{
        display "you need to include angnsf, anginc, angninc etc as first
argument"
        error 197
    }
    /*second argument linear or logit*/
    local linlog = "linear"
    if "`2'"=="logit" {
        local linlog = "logit"
    }
    local indname = "`1'"
    /*call ITSrandom*/
    qui ITSrandom `indname' `linlog'
    /*trans-QOF slope comparison with 2007*/
    qui lincom 2*slope_change - level_change
```

```
    scalar md1 = r(estimate)
    scalar lo1 = md1 - 1.96*r(se)
    scalar up1 = md1 + 1.96*r(se)
    scalar t = r(estimate)/r(se)
    qui count
    scalar df = r(N)-1
    scalar p1 = 2*ttail(df,abs(t))
    /*trans-QOF vs pre-QOF slope comparison*/
    qui lincom level_change
    scalar md2 = r(estimate)
    scalar lo2 = md2 - 1.96*r(se)
    scalar up2 = md2 + 1.96*r(se)
    scalar t = r(estimate)/r(se)
    qui count
    scalar df = r(N)-1
    scalar p2 = 2*ttail(df,abs(t))
    /*estimate of the QOF uplift in 07*/
    qui lincom level_change + 2*slope_change
    /*get estimated difference and confidence interval*/
    scalar ldiff_est = r(estimate)
    scalar ldiff_se = r(se)
    scalar ldiff_lo = ldiff_est - 1.96*ldiff_se
    scalar ldiff_up = ldiff_est + 1.96*ldiff_se
    /*get p-value*/
    scalar t = r(estimate)/r(se)
    qui count
    scalar df = r(N)-1
    scalar pval = 2*ttail(df,abs(t))
    /*get the observed mean of the logit transformed scores for 07 that
will serve as an "anchor" to calculate everything*/
    qui sum `indname'L if year==2007
    scalar obsL = r(mean)
    scalar predL = obsL - ldiff_est
    /*transform obs and pred to calculate the percentage difference*/
    scalar obsP = exp(obsL) / (1 + exp(obsL))
    scalar predP = exp(predL) / (1 + exp(predL))
    scalar diff_est = obsP - predP
    /*confidence intervals - option A, use obs score as base of
transformation*/
    scalar c1L = obsL - ldiff_lo
    scalar c2L = obsL - ldiff_up
    scalar c1 = exp(c1L) / (1 + exp(c1L))
    scalar c2 = exp(c2L) / (1 + exp(c2L))
    scalar diff_loA = obsP - c1
    scalar diff_upA = obsP - c2
    /*confidence intervals - option B, use pred score as base of
transformation*/
    scalar c1L = predL + ldiff_lo
    scalar c2L = predL + ldiff_up
    scalar c1 = exp(c1L) / (1 + exp(c1L))
    scalar c2 = exp(c2L) / (1 + exp(c2L))
    scalar diff_loB = c1-predP
    scalar diff_upB = c2-predP
    /*display results*/
    if "`linlog'"=="linear" {
        di _newline(2) "`indname' - `linlog' model" _col(40) "diff"
_col(50) "lowerCI" _col(60) "upperCI" _col(70) "p"
```

```
        di "pre-QOF estimate vs obs 07 (%)" _col(40) %5.3f ldiff_est*100
_col(50) %5.3f ldiff_lo*100 _col(60) %5.3f ldiff_up*100 _col(70) %5.3f
pval
        di "trans-QOF estimate vs obs 07 (%)" _col(40) %5.3f md1*100
_col(50) %5.3f lo1*100 _col(60) %5.3f up1*100 _col(70) %5.3f p1
        di "step change (%)" _col(40) %5.3f md2*100 _col(50) %5.3f
lo2*100 _col(60) %5.3f up2*100 _col(70) %5.3f p2
    }
    else {
        di _newline(2) "`indname' - `linlog' model" _col(40) "diff"
_col(50) "lowerCI" _col(60) "upperCI" _col(70) "p"
        di "pre-QOF estimate vs obs 07 (perc)" _col(40) %5.3f
diff_est*100 _col(50) %5.3f diff_loA*100 _col(60) %5.3f diff_upA*100
_col(70) %5.3f pval
        di _col(50) %5.3f diff_loB*100 _col(60) %5.3f diff_upB*100
        di "pre-QOF estimate vs obs 07 (logit)" _col(40) %5.3f ldiff_est
_col(50) %5.3f ldiff_lo _col(60) %5.3f ldiff_up _col(70) %5.3f pval
        di "trans-QOF estimate vs obs 07 (logit)" _col(40) %5.3f md1
_col(50) %5.3f lo1 _col(60) %5.3f up1 _col(70) %5.3f p1
        di "step change (logit)" _col(40) %5.3f md2 _col(50) %5.3f lo2
_col(60) %5.3f up2 _col(70) %5.3f p2
    }
end
```
