# Supplementary material for: Regression based quasi-experimental approach when randomisation is not an option: interrupted time series analysis
Source: BMJ. 2015 Jun 9;350:h2750. doi: 10.1136/bmj.h2750 (PMC4460815; doi:10.1136/bmj.h2750)
Supplement: Supplementary file 2 [file kone024026.ww2_default.pdf]

**Web appendix 2:**Code file for advanced ITS analysis

```
/*ITS analysis for patients in all time points*/
program mainanalysis
    local dir1 = "P:\Evan\GPRD\Diabetes\Analysis\"
    local dir2 = "P:\Evan\GPRD\Diabetes\Analysis\Logs\"
    cd "`dir1'"
    set more off
    tempfile temp
    /*get data*/
    qui use datafile1.dta, clear
    qui drop if year==1999 | year==2003
    /*generate variables needed for ITS approach*/
    /*make 2003-04 year 0*/
    qui gen cyear = year-2004
    /*dummy variable - binary intervention*/
    qui gen lvlchange = 0
    qui replace lvlchange = 1 if year>=2004
    /*dummy variable - interaction term - start counting AFTER 1st
intervention or post-intervention point*/
    /*after 1st time point where the intervention has had an effect*/
    qui gen slpchange = lvlchange*(year-2004)
    label var slpchange "Change in slope, post-contract"
    /*rename variables for better output*/
    qui rename cyear slppre

    if "`1'"=="linear" {
        local stradd=""
    }
    else if "`1'"=="logit" {
        local stradd="_logit"
    }
    else {
        di as err "logit or linear required"
        error 197
    }
    local sfx = ""
    if "`2'"!="" local sfx = "x"
    local indvar="DMall`sfx'`stradd'"
    /*set to the 0-100 scale if it's linear*/
    if "`1'"=="linear" {
        qui sum `indvar'
        if r(max)==1 {
            qui replace `indvar'=`indvar'*100
        }
    }
    qui drop if DMall==.
    qui drop if age<17

    /*standarsdise inputs for better interpretation of coefficients*/
    /*
    foreach xvar of varlist lvlchange slppre slpchange sex age
yearsofcare firstdiag comorb Angina-PAD IMDscore pracprev {
        qui sum `xvar'
        qui replace `xvar' = (`xvar'-r(mean))/(2*r(sd))
    } */
```

```
    /*log output*/
    qui log using "`dir2'2-ITS2lvl_`1'`sfx'", name(log1) smcl replace

    /*collinearity test*/
    *collin lvlchange slppre slpchange sex age yearsofcare diagyear
firstfullyear comorb DMall`sfx'count IMDscore pracprev

    di _newline(2) "only using patients aged 17 or over"
    if "`sfx'" == "x" {
        di "Indicators DM3 and DM4 have been excluded from the analysis"
    }

    /*model testing for differences in the pre-QOF era*/
    di _newline(2) "Model testing for differences between groups in the
pre-QOF era"
    xtmixed `indvar' i.sex i.agecat i.yocc i.comorb i.IMDscorecat i.ppc
if year<=2002 || praceid: || pateid:
    test 1.agecat=2.agecat=0
    test 1.yocc= 2.yocc= 3.yocc=0
    test 1.comorb=2.comorb=3.comorb=0
    test 1.IMDscorecat=2.IMDscorecat=3.IMDscorecat=0
    test 1.ppc= 2.ppc=3.ppc=0
    /*back transformations*/
    if "`1'"=="logit" {
        /*since we only have the difference we need to calculate the
observed score across the three years and base the back-transforamtion on
that*/
        qui sum DMall_logit /*if year<=2002*/
        scalar lgscorePRE = r(mean)
        /*loop across all variables*/
        di _newline(2) "Back-transformed" _col(25) "eff" _col(35) "L95%"
_col(45) "U95%" _col(55) "pval"
        foreach x in 1.sex 1.agecat 2.agecat 1.yocc 2.yocc 3.yocc
1.comorb 2.comorb 3.comorb 1.IMDscorecat 2.IMDscorecat /*
        */ 3.IMDscorecat 1.ppc 2.ppc 3.ppc {
            scalar yxb = _b[`x']
            scalar ylb = _b[`x']-1.96*_se[`x']
            scalar yub = _b[`x']+1.96*_se[`x']
            /*calculate p-value*/
            scalar tempse = (yub - yxb)/1.96
            scalar tempz = yxb/tempse
            scalar tempp = 2*(1-normal(abs(tempz)))
            /*back transforming*/
            /*using raw overall mean and difference to get "observed"
score (for eff AND CIs)*/
            scalar teff = lgscorePRE+yxb
            scalar tl95 = lgscorePRE+ylb
            scalar tu95 = lgscorePRE+yub
            /*backtrasnformations to percentages*/
            scalar bt1 = 100*1/(1+exp(-lgscorePRE))
            scalar bteff = 100*1/(1+exp(-teff))
            scalar btl95 = 100*1/(1+exp(-tl95))
            scalar btu95 = 100*1/(1+exp(-tu95))
            /*and differences on back-transformations ("observed" -
predicted)*/
            scalar deff = bteff-bt1
            scalar dl95 = btl95-bt1
```

```
            scalar du95 = btu95-bt1
            if "`x'"=="2.ppc" {
                di "`x'" _col(25) "%" %4.2f deff _col(35) "%" %4.1f dl95
_col(45) "%" %4.1f du95 _col(55) %5.3f tempp
            }
            else {
                di "`x'" _col(25) "%" %4.1f deff _col(35) "%" %4.1f dl95
_col(45) "%" %4.1f du95 _col(55) %5.3f tempp
            }
        }
    }

    /*model testing for differences in the post-QOF era*/
    di _newline(2) "Model testing for differences between groups in the
post-QOF era"
    xtmixed `indvar' i.sex i.agecat i.yocc i.comorb i.IMDscorecat i.ppc
if year>=2004 || praceid: || pateid:
    test 1.agecat=2.agecat=0
    test 1.yocc= 2.yocc= 3.yocc=0
    test 1.comorb=2.comorb=3.comorb=0
    test 1.IMDscorecat=2.IMDscorecat=3.IMDscorecat=0
    test 1.ppc=2.ppc=3.ppc=0
    /*back transformations*/
    if "`1'"=="logit" {
        /*since we only have the difference we need to calculate the
observed score across the three years and base the back-transforamtion on
that*/
        qui sum DMall_logit /*if year>=2004*/
        scalar lgscorePRE = r(mean)
        /*loop across all variables*/
        di _newline(2) "Back-transformed" _col(25) "eff" _col(35) "L95%"
_col(45) "U95%" _col(55) "pval"
        foreach x in 1.sex 1.agecat 2.agecat 1.yocc 2.yocc 3.yocc
1.comorb 2.comorb 3.comorb 1.IMDscorecat 2.IMDscorecat /*
        */ 3.IMDscorecat 1.ppc 2.ppc 3.ppc {
            scalar yxb = _b[`x']
            scalar ylb = _b[`x']-1.96*_se[`x']
            scalar yub = _b[`x']+1.96*_se[`x']
            /*calculate p-value*/
            scalar tempse = (yub - yxb)/1.96
            scalar tempz = yxb/tempse
            scalar tempp = 2*(1-normal(abs(tempz)))
            /*back transforming*/
            /*using raw overall mean and difference to get "observed"
score (for eff AND CIs)*/
            scalar teff = lgscorePRE+yxb
            scalar tl95 = lgscorePRE+ylb
            scalar tu95 = lgscorePRE+yub
            /*backtrasnformations to percentages*/
            scalar bt1 = 100*1/(1+exp(-lgscorePRE))
            scalar bteff = 100*1/(1+exp(-teff))
            scalar btl95 = 100*1/(1+exp(-tl95))
            scalar btu95 = 100*1/(1+exp(-tu95))
            /*and differences on back-transformations ("observed" -
predicted)*/
            scalar deff = bteff-bt1
            scalar dl95 = btl95-bt1
            scalar du95 = btu95-bt1
```

```
            if "`x'"=="2.IMDscorecat" {
                di "`x'" _col(25) "%" %4.1f deff _col(35) "%" %4.1f dl95
_col(45) "%" %4.2f du95 _col(55) %5.3f tempp
            }
            else {
                di "`x'" _col(25) "%" %4.1f deff _col(35) "%" %4.1f dl95
_col(45) "%" %4.1f du95 _col(55) %5.3f tempp
            }

        }
    }

    /*MAIN model - not including any patient-level variables since the
analysis won't be controlled; only an estimate of the overall effect
across time,
    for each variable, will be received */
    di _newline(2) "Main model"
    xtmixed `indvar' lvlchange slppre slpchange || praceid: || pateid:
    /*To get CIs from the post tests you can take the square root of the
chi-square value to get a z-value, then divide the coef
    by this to get a value for the SE and use coef +/- (SE x 1.96) to
give the plus/minus 95% CI*/
    /*post-test: compare pre-qof extrapolated trend to true scores in0
the last time point*/
    di _newline(1) in yellow "Comparing pre-qof extrapolated trend to
true scores in 06-07"
    test lvlchange + 2*slpchange = 0    /*lvl change over 2 years*/
    scalar df = 100000
    scalar zval = sqrt(r(chi2))
    scalar up0607xb = _b[lvlchange] + 2*_b[slpchange]
    scalar seval = up0607xb/zval
    scalar up0607lb = up0607xb - invttail(df,0.025)*seval
    scalar up0607ub = up0607xb + invttail(df,0.025)*seval
    di  _col(10) "zval = " %4.2f zval
    di _newline(1) _col(15) "95% Conf. Interval"
    di _col(5) "Coeff" _col(15) "Lower" _col(25) "Upper"
    di _col(5) %5.4f up0607xb _col(15) %5.4f up0607lb _col(25) %5.4f
up0607ub
    /*post-test: post trend (i.e. 2006/07 vs 2004/05) */
    di _newline(1) in yellow "Comparing 04-05 scores to 06-07 (slope)"
    test slppre + slpchange = 0
    scalar df = 100000
    scalar zval = sqrt(r(chi2))
    scalar changexb = _b[slppre] + _b[slpchange]
    scalar seval = changexb/zval
    scalar changelb = changexb - invttail(df,0.025)*seval
    scalar changeub = changexb + invttail(df,0.025)*seval
    di  _col(10) "zval = " %4.2f zval
    di _newline(1) _col(15) "95% Conf. Interval"
    di _col(5) "Coeff" _col(15) "Lower" _col(25) "Upper"
    di _col(5) %5.4f changexb _col(15) %5.4f changelb _col(25) %5.4f
changeub
    /*back transformations*/
    if "`1'"=="logit" {
        /*since we only have the difference we need to calculate the
observed score and base the back-transforamtion on that*/
        forvalues i=2000(1)2006 {
            qui sum DMall_logit if year==`i'
```

```
            scalar lgscore`i' = r(mean)
        }
        scalar RMchange = lgscore2006-lgscore2004
        scalar RMup0405 = (11*lgscore2002+2*lgscore2001-7*lgscore2000)/6
        scalar RMup0607 = (17*lgscore2002+2*lgscore2001-13*lgscore2000)/6
        scalar up0405xb = _b[lvlchange]
        scalar up0405lb = _b[lvlchange]-1.96*_se[lvlchange]
        scalar up0405ub = _b[lvlchange]+1.96*_se[lvlchange]
        di _newline(2) "Back-transformed" _col(25) "eff" _col(35) "L95%"
_col(45) "U95%" _col(55) "pval"
        foreach outc in up0405 up0607 change {
            /*calculate p-value*/
            scalar tempse = (`outc'ub - `outc'xb)/1.96
            scalar tempz = `outc'xb/tempse
            scalar tempp = 2*(1-normal(abs(tempz)))
            /*back transforming*/
            /*using raw predicted overall mean and difference to get
"observed" score (for eff AND CIs)*/
            scalar teff = RM`outc'+`outc'xb
            scalar tl95 = RM`outc'+`outc'lb
            scalar tu95 = RM`outc'+`outc'ub
            /*backtrasnformations to percentages*/
            scalar bt1 = 100*1/(1+exp(-RM`outc'))
            scalar bteff = 100*1/(1+exp(-teff))
            scalar btl95 = 100*1/(1+exp(-tl95))
            scalar btu95 = 100*1/(1+exp(-tu95))
            /*and differences on back-transformations ("observed" -
predicted)*/
            scalar deff = bteff-bt1
            scalar dl95 = btl95-bt1
            scalar du95 = btu95-bt1
            di "`outc'" _col(25) "%" %4.1f deff _col(35) "%" %4.1f dl95
_col(45) "%" %4.1f du95 _col(55) %5.3f tempp
        }
    }

    /*INTERACTIONS*/
    /*GENDER CATEGORY*/
    capture drop _*
    di _newline(2)
    xi: xtmixed `indvar' i.sex*lvlchange i.sex*slppre i.sex*slpchange ||
praceid: || pateid:
    di _newline(1) in yellow "Comparing pre-qof extrapolated trend
differences to true score differences in 06-07"
    forvalues i=1(1)1 {
        di _newline(1) in yellow "Female (=1) vs male (=0)"
        test _IsexXlvlch_`i' + 2*_IsexXslpch_`i' = 0
        scalar df = 100000
        scalar zval = sqrt(r(chi2))
        scalar up0607xb`i' = _b[_IsexXlvlch_`i'] + 2*_b[_IsexXslpch_`i']
        scalar seval = up0607xb`i'/zval
        scalar up0607lb`i' = up0607xb`i' - invttail(df,0.025)*seval
        scalar up0607ub`i' = up0607xb`i' + invttail(df,0.025)*seval
        di  _col(10) "zval = " %4.2f zval
        di _newline(1) _col(15) "95% Conf. Interval"
        di _col(5) "Coeff" _col(15) "Lower" _col(25) "Upper"
        di _col(5) %5.4f up0607xb`i' _col(15) %5.4f up0607lb`i' _col(25)
%5.4f up0607ub`i'
```

```
    }
    di _newline(1) in yellow "Comparing 04-05 scores to 06-07 (slope)"
    forvalues i=1(1)1 {
        di _newline(1) in yellow "Female (=1) vs male (=0)"
        test _IsexXslppr_`i' + _IsexXslpch_`i' = 0
        scalar df = 100000
        scalar zval = sqrt(r(chi2))
        scalar changexb`i' = _b[_IsexXslppr_`i'] + _b[_IsexXslpch_`i']
        scalar seval = changexb`i'/zval
        scalar changelb`i' = changexb`i' - invttail(df,0.025)*seval
        scalar changeub`i' = changexb`i' + invttail(df,0.025)*seval
        di  _col(10) "zval = " %4.2f zval
        di _newline(1) _col(15) "95% Conf. Interval"
        di _col(5) "Coeff" _col(15) "Lower" _col(25) "Upper"
        di _col(5) %5.4f changexb`i' _col(15) %5.4f changelb`i' _col(25)
%5.4f changeub`i'
    }
    /*back transformations (some of the scalars used here were calculated
in the back-trans for the main model)*/
    if "`1'"=="logit" {
        forvalues i=1(1)1 {
            scalar up0405xb`i' = _b[_IsexXlvlch_`i']
            scalar up0405lb`i' = _b[_IsexXlvlch_`i']-
1.96*_se[_IsexXlvlch_`i']
            scalar up0405ub`i' =
_b[_IsexXlvlch_`i']+1.96*_se[_IsexXlvlch_`i']
            di _newline(2) "Back-tr Sex cat `i'" _col(25) "eff" _col(35)
"L95%" _col(45) "U95%" _col(55) "pval"
            foreach outc in up0405 up0607 change {
                /*calculate p-value*/
                scalar tempse = (`outc'ub`i' - `outc'xb`i')/1.96
                scalar tempz = `outc'xb`i'/tempse
                scalar tempp = 2*(1-normal(abs(tempz)))
                /*back transforming*/
                /*using raw predicted overall mean and difference to get
"observed" score (for eff AND CIs)*/
                scalar teff = RM`outc'+`outc'xb`i'
                scalar tl95 = RM`outc'+`outc'lb`i'
                scalar tu95 = RM`outc'+`outc'ub`i'
                /*backtrasnformations to percentages*/
                scalar bt1 = 100*1/(1+exp(-RM`outc'))
                scalar bteff = 100*1/(1+exp(-teff))
                scalar btl95 = 100*1/(1+exp(-tl95))
                scalar btu95 = 100*1/(1+exp(-tu95))
                /*and differences on back-transformations ("observed" -
predicted)*/
                scalar deff = bteff-bt1
                scalar dl95 = btl95-bt1
                scalar du95 = btu95-bt1
                if `i'==1 & "`outc'"=="up0607" {
                    di "`outc'" _col(25) "%" %4.1f deff _col(35) "%"
%4.2f dl95 _col(45) "%" %4.1f du95 _col(55) %5.3f tempp
                }
                else {
                    di "`outc'" _col(25) "%" %4.1f deff _col(35) "%"
%4.1f dl95 _col(45) "%" %4.1f du95 _col(55) %5.3f tempp
                }
            }
```

```
        }
    }

    /*AGE CATEGORY*/
    capture drop _*
    di _newline(2)
    xi: xtmixed `indvar' i.agecat*lvlchange i.agecat*slppre
i.agecat*slpchange || praceid: || pateid:
    /***/
    di _newline(1) in yellow "Comparing pre-qof extrapolated trend
differences to true score differences in 04-05"
    di _newline(1) in yellow "Overall across all ages"
    test _IageXlvlch_1 = _IageXlvlch_2 = 0
    di _newline(1) in yellow "Comparing pre-qof extrapolated trend
differences to true score differences in 06-07"
    forvalues i=1(1)2 {
        di _newline(1) in yellow "Age cat `i' vs 0"
        test _IageXlvlch_`i' + 2*_IageXslpch_`i' = 0
        scalar df = 100000
        scalar zval = sqrt(r(chi2))
        scalar up0607xb`i' = _b[_IageXlvlch_`i'] + 2*_b[_IageXslpch_`i']
        scalar seval = up0607xb`i'/zval
        scalar up0607lb`i' = up0607xb`i' - invttail(df,0.025)*seval
        scalar up0607ub`i' = up0607xb`i' + invttail(df,0.025)*seval
        di  _col(10) "zval = " %4.2f zval
        di _newline(1) _col(15) "95% Conf. Interval"
        di _col(5) "Coeff" _col(15) "Lower" _col(25) "Upper"
        di _col(5) %5.4f up0607xb`i' _col(15) %5.4f up0607lb`i' _col(25)
%5.4f up0607ub`i'
    }
    di _newline(1) in yellow "Overall across all ages"
    test _IageXlvlch_1 + 2*_IageXslpch_1 = _IageXlvlch_2 +
2*_IageXslpch_2 = 0
    /***/
    di _newline(1) in yellow "Comparing 04-05 scores to 06-07 (slope)"
    forvalues i=1(1)2 {
        di _newline(1) in yellow "Age cat `i' vs 0"
        test _IageXslppr_`i' + _IageXslpch_`i' = 0
        scalar df = 100000
        scalar zval = sqrt(r(chi2))
        scalar changexb`i' = _b[_IageXslppr_`i'] + _b[_IageXslpch_`i']
        scalar seval = changexb`i'/zval
        scalar changelb`i' = changexb`i' - invttail(df,0.025)*seval
        scalar changeub`i' = changexb`i' + invttail(df,0.025)*seval
        di  _col(10) "zval = " %4.2f zval
        di _newline(1) _col(15) "95% Conf. Interval"
        di _col(5) "Coeff" _col(15) "Lower" _col(25) "Upper"
        di _col(5) %5.4f changexb`i' _col(15) %5.4f changelb`i' _col(25)
%5.4f changeub`i'
    }
    di _newline(1) in yellow "Overall across all ages"
    test _IageXslppr_1 + _IageXslpch_1 = _IageXslppr_2 + _IageXslpch_2 =
0
    /*back transformations (some of the scalars used here were calculated
in the back-trans for the main model)*/
    if "`1'"=="logit" {
        forvalues i=1(1)2 {
            scalar up0405xb`i' = _b[_IageXlvlch_`i']
```

```
            scalar up0405lb`i' = _b[_IageXlvlch_`i']-
1.96*_se[_IageXlvlch_`i']
            scalar up0405ub`i' =
_b[_IageXlvlch_`i']+1.96*_se[_IageXlvlch_`i']
            di _newline(2) "Back-tr Age cat `i'" _col(25) "eff" _col(35)
"L95%" _col(45) "U95%" _col(55) "pval"
            foreach outc in up0405 up0607 change {
                /*calculate p-value*/
                scalar tempse = (`outc'ub`i' - `outc'xb`i')/1.96
                scalar tempz = `outc'xb`i'/tempse
                scalar tempp = 2*(1-normal(abs(tempz)))
                /*back transforming*/
                /*using raw predicted overall mean and difference to get
"observed" score (for eff AND CIs)*/
                scalar teff = RM`outc'+`outc'xb`i'
                scalar tl95 = RM`outc'+`outc'lb`i'
                scalar tu95 = RM`outc'+`outc'ub`i'
                /*backtrasnformations to percentages*/
                scalar bt1 = 100*1/(1+exp(-RM`outc'))
                scalar bteff = 100*1/(1+exp(-teff))
                scalar btl95 = 100*1/(1+exp(-tl95))
                scalar btu95 = 100*1/(1+exp(-tu95))
                /*and differences on back-transformations ("observed" -
predicted)*/
                scalar deff = bteff-bt1
                scalar dl95 = btl95-bt1
                scalar du95 = btu95-bt1
                if `i'==1 & "`outc'"=="up0607" {
                    di "`outc'" _col(25) "%" %4.1f deff _col(35) "%"
%4.1f dl95 _col(45) "%" %4.1f du95 _col(55) %5.3f tempp
                }
                else {
                    di "`outc'" _col(25) "%" %4.1f deff _col(35) "%"
%4.1f dl95 _col(45) "%" %4.1f du95 _col(55) %5.3f tempp
                }
            }
        }
    }

    /*YEARS OF CARE CATEGORICAL*/
    capture drop _*
    di _newline(2)
    xi: xtmixed `indvar' i.yocc*lvlchange i.yocc*slppre i.yocc*slpchange
|| praceid: || pateid:
    /***/
    di _newline(1) in yellow "Comparing pre-qof extrapolated trend
differences to true score differences in 04-05"
    di _newline(1) in yellow "Overall across all years of care
categories"
    test _IyocXlvlch_1 = _IyocXlvlch_2 = _IyocXlvlch_3 = 0
    di _newline(1) in yellow "Comparing pre-qof extrapolated trend
differences to true score differences in 06-07"
    forvalues i=1(1)3 {
        di _newline(1) in yellow "Years of care cat `i' vs 0"
        test _IyocXlvlch_`i' + 2*_IyocXslpch_`i' = 0
        scalar df = 100000
        scalar zval = sqrt(r(chi2))
        scalar up0607xb`i' = _b[_IyocXlvlch_`i'] + 2*_b[_IyocXslpch_`i']
```

```
        scalar seval = up0607xb`i'/zval
        scalar up0607lb`i' = up0607xb`i' - invttail(df,0.025)*seval
        scalar up0607ub`i' = up0607xb`i' + invttail(df,0.025)*seval
        di  _col(10) "zval = " %4.2f zval
        di _newline(1) _col(15) "95% Conf. Interval"
        di _col(5) "Coeff" _col(15) "Lower" _col(25) "Upper"
        di _col(5) %5.4f up0607xb`i' _col(15) %5.4f up0607lb`i' _col(25)
%5.4f up0607ub`i'
    }
    di _newline(1) in yellow "Overall across all years of care
categories"
    test _IyocXlvlch_1 + 2*_IyocXslpch_1 = _IyocXlvlch_2 +
2*_IyocXslpch_2 = _IyocXlvlch_3 + 2*_IyocXslpch_3 = 0
    /***/
    di _newline(1) in yellow "Comparing 04-05 scores to 06-07 (slope)"
    forvalues i=1(1)3 {
        di _newline(1) in yellow "Years of care cat `i' vs 0"
        test _IyocXslppr_`i' + _IyocXslpch_`i' = 0
        scalar df = 100000
        scalar zval = sqrt(r(chi2))
        scalar changexb`i' = _b[_IyocXslppr_`i'] + _b[_IyocXslpch_`i']
        scalar seval = changexb`i'/zval
        scalar changelb`i' = changexb`i' - invttail(df,0.025)*seval
        scalar changeub`i' = changexb`i' + invttail(df,0.025)*seval
        di  _col(10) "zval = " %4.2f zval
        di _newline(1) _col(15) "95% Conf. Interval"
        di _col(5) "Coeff" _col(15) "Lower" _col(25) "Upper"
        di _col(5) %5.4f changexb`i' _col(15) %5.4f changelb`i' _col(25)
%5.4f changeub`i'
    }
    di _newline(1) in yellow "Overall across all years of care
categories"
    test _IyocXslppr_1 + _IyocXslpch_1 = _IyocXslppr_2 + _IyocXslpch_2 =
_IyocXslppr_3 + _IyocXslpch_3 = 0
    /*back transformations (some of the scalars used here were calculated
in the back-trans for the main model)*/
    if "`1'"=="logit" {
        forvalues i=1(1)3 {
            scalar up0405xb`i' = _b[_IyocXlvlch_`i']
            scalar up0405lb`i' = _b[_IyocXlvlch_`i']-
1.96*_se[_IyocXlvlch_`i']
            scalar up0405ub`i' =
_b[_IyocXlvlch_`i']+1.96*_se[_IyocXlvlch_`i']
            di _newline(2) "Back-tr YOC cat `i'" _col(25) "eff" _col(35)
"L95%" _col(45) "U95%" _col(55) "pval"
            foreach outc in up0405 up0607 change {
                /*calculate p-value*/
                scalar tempse = (`outc'ub`i' - `outc'xb`i')/1.96
                scalar tempz = `outc'xb`i'/tempse
                scalar tempp = 2*(1-normal(abs(tempz)))
                /*back transforming*/
                /*using raw predicted overall mean and difference to get
"observed" score (for eff AND CIs)*/
                scalar teff = RM`outc'+`outc'xb`i'
                scalar tl95 = RM`outc'+`outc'lb`i'
                scalar tu95 = RM`outc'+`outc'ub`i'
                /*backtrasnformations to percentages*/
                scalar bt1 = 100*1/(1+exp(-RM`outc'))
```

```
                scalar bteff = 100*1/(1+exp(-teff))
                scalar btl95 = 100*1/(1+exp(-tl95))
                scalar btu95 = 100*1/(1+exp(-tu95))
                /*and differences on back-transformations ("observed" -
predicted)*/
                scalar deff = bteff-bt1
                scalar dl95 = btl95-bt1
                scalar du95 = btu95-bt1
                if `i'==1 & "`outc'"=="up0607" {
                    di "`outc'" _col(25) "%" %4.1f deff _col(35) "%"
%4.1f dl95 _col(45) "%" %4.1f du95 _col(55) %5.3f tempp
                }
                else {
                    di "`outc'" _col(25) "%" %4.1f deff _col(35) "%"
%4.1f dl95 _col(45) "%" %4.1f du95 _col(55) %5.3f tempp
                }
            }
        }
    }

    /*NUMBER OF CO-MORBIDITIES*/
    capture drop _*
    di _newline(2)
    xi: xtmixed `indvar' i.comorb*lvlchange i.comorb*slppre
i.comorb*slpchange || praceid: || pateid:
    /***/
    di _newline(1) in yellow "Comparing pre-qof extrapolated trend
differences to true score differences in 04-05"
    di _newline(1) in yellow "Overall across all comorbidities
categories"
    test _IcomXlvlch_1 = _IcomXlvlch_2 = _IcomXlvlch_3 = 0
    di _newline(1) in yellow "Comparing pre-qof extrapolated trend
differences to true score differences in 06-07"
    forvalues i=1(1)3 {
        di _newline(1) in yellow "# of comorbidities cat `i' vs 0"
        test _IcomXlvlch_`i' + 2*_IcomXslpch_`i' = 0
        scalar df = 100000
        scalar zval = sqrt(r(chi2))
        scalar up0607xb`i' = _b[_IcomXlvlch_`i'] + 2*_b[_IcomXslpch_`i']
        scalar seval = up0607xb`i'/zval
        scalar up0607lb`i' = up0607xb`i' - invttail(df,0.025)*seval
        scalar up0607ub`i' = up0607xb`i' + invttail(df,0.025)*seval
        di  _col(10) "zval = " %4.2f zval
        di _newline(1) _col(15) "95% Conf. Interval"
        di _col(5) "Coeff" _col(15) "Lower" _col(25) "Upper"
        di _col(5) %5.4f up0607xb`i' _col(15) %5.4f up0607lb`i' _col(25)
%5.4f up0607ub`i'
    }
    di _newline(1) in yellow "Overall across all comorbidities
categories"
    test _IcomXlvlch_1 + 2*_IcomXslpch_1 = _IcomXlvlch_2 +
2*_IcomXslpch_2 = _IcomXlvlch_3 + 2*_IcomXslpch_3 = 0
    /***/
    di _newline(1) in yellow "Comparing 04-05 scores to 06-07 (slope)"
    forvalues i=1(1)3 {
        di _newline(1) in yellow "# of comorbidities cat `i' vs 0"
        test _IcomXslppr_`i' + _IcomXslpch_`i' = 0
        scalar df = 100000
```

```
        scalar zval = sqrt(r(chi2))
        scalar changexb`i' = _b[_IcomXslppr_`i'] + _b[_IcomXslpch_`i']
        scalar seval = changexb`i'/zval
        scalar changelb`i' = changexb`i' - invttail(df,0.025)*seval
        scalar changeub`i' = changexb`i' + invttail(df,0.025)*seval
        di  _col(10) "zval = " %4.2f zval
        di _newline(1) _col(15) "95% Conf. Interval"
        di _col(5) "Coeff" _col(15) "Lower" _col(25) "Upper"
        di _col(5) %5.4f changexb`i' _col(15) %5.4f changelb`i' _col(25)
%5.4f changeub`i'
    }
    di _newline(1) in yellow "Overall across all comorbidities
categories"
    test _IcomXslppr_1 + _IcomXslpch_1 = _IcomXslppr_2 + _IcomXslpch_2 =
_IcomXslppr_3 + _IcomXslpch_3 = 0
    /*back transformations (some of the scalars used here were calculated
in the back-trans for the main model)*/
    if "`1'"=="logit" {
        forvalues i=1(1)3 {
            scalar up0405xb`i' = _b[_IcomXlvlch_`i']
            scalar up0405lb`i' = _b[_IcomXlvlch_`i']-
1.96*_se[_IcomXlvlch_`i']
            scalar up0405ub`i' =
_b[_IcomXlvlch_`i']+1.96*_se[_IcomXlvlch_`i']
            di _newline(2) "Back-tr Comorb cat `i'" _col(25) "eff"
_col(35) "L95%" _col(45) "U95%" _col(55) "pval"
            foreach outc in up0405 up0607 change {
                /*calculate p-value*/
                scalar tempse = (`outc'ub`i' - `outc'xb`i')/1.96
                scalar tempz = `outc'xb`i'/tempse
                scalar tempp = 2*(1-normal(abs(tempz)))
                /*back transforming*/
                /*using raw predicted overall mean and difference to get
"observed" score (for eff AND CIs)*/
                scalar teff = RM`outc'+`outc'xb`i'
                scalar tl95 = RM`outc'+`outc'lb`i'
                scalar tu95 = RM`outc'+`outc'ub`i'
                /*backtrasnformations to percentages*/
                scalar bt1 = 100*1/(1+exp(-RM`outc'))
                scalar bteff = 100*1/(1+exp(-teff))
                scalar btl95 = 100*1/(1+exp(-tl95))
                scalar btu95 = 100*1/(1+exp(-tu95))
                /*and differences on back-transformations ("observed" -
predicted)*/
                scalar deff = bteff-bt1
                scalar dl95 = btl95-bt1
                scalar du95 = btu95-bt1
                if `i'==1 & "`outc'"=="up0607" {
                    di "`outc'" _col(25) "%" %4.1f deff _col(35) "%"
%4.1f dl95 _col(45) "%" %4.1f du95 _col(55) %5.3f tempp
                }
                else {
                    di "`outc'" _col(25) "%" %4.1f deff _col(35) "%"
%4.1f dl95 _col(45) "%" %4.1f du95 _col(55) %5.3f tempp
                }
            }
        }
    }
```

```
    /* IMD score category*/
    capture drop _*
    di _newline(2)
    xi: xtmixed `indvar' i.IMDscorecat*lvlchange i.IMDscorecat*slppre
i.IMDscorecat*slpchange || praceid: || pateid:
    /***/
    di _newline(1) in yellow "Comparing pre-qof extrapolated trend
differences to true score differences in 04-05"
    di _newline(1) in yellow "Overall across all IMD categories"
    test _IIMDXlvlch_1 = _IIMDXlvlch_2 = _IIMDXlvlch_3 = 0
    di _newline(1) in yellow "Comparing pre-qof extrapolated trend
differences to true score differences in 06-07"
    forvalues i=1(1)3 {
        di _newline(1) in yellow "IMD cat lvl `i' vs 0"
        test _IIMDXlvlch_`i' + 2*_IIMDXslpch_`i' = 0
        scalar df = 100000
        scalar zval = sqrt(r(chi2))
        scalar up0607xb`i' = _b[_IIMDXlvlch_`i'] + 2*_b[_IIMDXslpch_`i']
        scalar seval = up0607xb`i'/zval
        scalar up0607lb`i' = up0607xb`i' - invttail(df,0.025)*seval
        scalar up0607ub`i' = up0607xb`i' + invttail(df,0.025)*seval
        di  _col(10) "zval = " %4.2f zval
        di _newline(1) _col(15) "95% Conf. Interval"
        di _col(5) "Coeff" _col(15) "Lower" _col(25) "Upper"
        di _col(5) %5.4f up0607xb`i' _col(15) %5.4f up0607lb`i' _col(25)
%5.4f up0607ub`i'
    }
    di _newline(1) in yellow "Overall across all IMD categories"
    test _IIMDXlvlch_1 + 2*_IIMDXslpch_1 = _IIMDXlvlch_2 +
2*_IIMDXslpch_2 = _IIMDXlvlch_3 + 2*_IIMDXslpch_3 = 0
    /***/
    di _newline(1) in yellow "Comparing 04-05 scores to 06-07 (slope)"
    forvalues i=1(1)3 {
        di _newline(1) in yellow "IMD cat lvl `i' vs 0"
        test _IIMDXslppr_`i' + _IIMDXslpch_`i' = 0
        scalar df = 100000
        scalar zval = sqrt(r(chi2))
        scalar changexb`i' = _b[_IIMDXslppr_`i'] + _b[_IIMDXslpch_`i']
        scalar seval = changexb`i'/zval
        scalar changelb`i' = changexb`i' - invttail(df,0.025)*seval
        scalar changeub`i' = changexb`i' + invttail(df,0.025)*seval
        di  _col(10) "zval = " %4.2f zval
        di _newline(1) _col(15) "95% Conf. Interval"
        di _col(5) "Coeff" _col(15) "Lower" _col(25) "Upper"
        di _col(5) %5.4f changexb`i' _col(15) %5.4f changelb`i' _col(25)
%5.4f changeub`i'
    }
    di _newline(1) in yellow "Overall across all IMD categories"
    test _IIMDXslppr_1 + _IIMDXslpch_1 = _IIMDXslppr_2 + _IIMDXslpch_2 =
_IIMDXslppr_3 + _IIMDXslpch_3 = 0
    /*back transformations (some of the scalars used here were calculated
in the back-trans for the main model)*/
    if "`1'"=="logit" {
        forvalues i=1(1)3 {
            scalar up0405xb`i' = _b[_IIMDXlvlch_`i']
            scalar up0405lb`i' = _b[_IIMDXlvlch_`i']-
1.96*_se[_IIMDXlvlch_`i']
```

```
            scalar up0405ub`i' =
_b[_IIMDXlvlch_`i']+1.96*_se[_IIMDXlvlch_`i']
            di _newline(2) "Back-tr IMD cat `i'" _col(25) "eff" _col(35)
"L95%" _col(45) "U95%" _col(55) "pval"
            foreach outc in up0405 up0607 change {
                /*calculate p-value*/
                scalar tempse = (`outc'ub`i' - `outc'xb`i')/1.96
                scalar tempz = `outc'xb`i'/tempse
                scalar tempp = 2*(1-normal(abs(tempz)))
                /*back transforming*/
                /*using raw predicted overall mean and difference to get
"observed" score (for eff AND CIs)*/
                scalar teff = RM`outc'+`outc'xb`i'
                scalar tl95 = RM`outc'+`outc'lb`i'
                scalar tu95 = RM`outc'+`outc'ub`i'
                /*backtrasnformations to percentages*/
                scalar bt1 = 100*1/(1+exp(-RM`outc'))
                scalar bteff = 100*1/(1+exp(-teff))
                scalar btl95 = 100*1/(1+exp(-tl95))
                scalar btu95 = 100*1/(1+exp(-tu95))
                /*and differences on back-transformations ("observed" -
predicted)*/
                scalar deff = bteff-bt1
                scalar dl95 = btl95-bt1
                scalar du95 = btu95-bt1
                if `i'==1 & "`outc'"=="up0607" {
                    di "`outc'" _col(25) "%" %4.1f deff _col(35) "%"
%4.1f dl95 _col(45) "%" %4.1f du95 _col(55) %5.3f tempp
                }
                else {
                    di "`outc'" _col(25) "%" %4.1f deff _col(35) "%"
%4.1f dl95 _col(45) "%" %4.1f du95 _col(55) %5.3f tempp
                }
            }
        }
    }

    /*practice prevalence*/
    capture drop _*
    di _newline(2)
    xi: xtmixed `indvar' i.ppc*lvlchange i.ppc*slppre i.ppc*slpchange ||
praceid: || pateid:
    /***/
    di _newline(1) in yellow "Comparing pre-qof extrapolated trend
differences to true score differences in 04-05"
    di _newline(1) in yellow "Overall across all prevalence categories"
    test _IppcXlvlch_1 = _IppcXlvlch_2 = _IppcXlvlch_3 = 0
    di _newline(1) in yellow "Comparing pre-qof extrapolated trend
differences to true score differences in 06-07"
    forvalues i=1(1)3 {
        di _newline(1) in yellow "Prevalence cat `i' vs 0"
        test _IppcXlvlch_`i' + 2*_IppcXslpch_`i' = 0
        scalar df = 100000
        scalar zval = sqrt(r(chi2))
        scalar up0607xb`i' = _b[_IppcXlvlch_`i'] + 2*_b[_IppcXslpch_`i']
        scalar seval = up0607xb`i'/zval
        scalar up0607lb`i' = up0607xb`i' - invttail(df,0.025)*seval
        scalar up0607ub`i' = up0607xb`i' + invttail(df,0.025)*seval
```

```
        di  _col(10) "zval = " %4.2f zval
        di _newline(1) _col(15) "95% Conf. Interval"
        di _col(5) "Coeff" _col(15) "Lower" _col(25) "Upper"
        di _col(5) %5.4f up0607xb`i' _col(15) %5.4f up0607lb`i' _col(25)
%5.4f up0607ub`i'
    }
    di _newline(1) in yellow "Overall across all prevalence categories"
    test _IppcXlvlch_1 + 2*_IppcXslpch_1 = _IppcXlvlch_2 +
2*_IppcXslpch_2 = _IppcXlvlch_3 + 2*_IppcXslpch_3 = 0
    /***/
    di _newline(1) in yellow "Comparing 04-05 scores to 06-07 (slope)"
    forvalues i=1(1)3 {
        di _newline(1) in yellow "Prevalence cat `i' vs 0"
        test _IppcXslppr_`i' + _IppcXslpch_`i' = 0
        scalar df = 100000
        scalar zval = sqrt(r(chi2))
        scalar changexb`i' = _b[_IppcXslppr_`i'] + _b[_IppcXslpch_`i']
        scalar seval = changexb`i'/zval
        scalar changelb`i' = changexb`i' - invttail(df,0.025)*seval
        scalar changeub`i' = changexb`i' + invttail(df,0.025)*seval
        di  _col(10) "zval = " %4.2f zval
        di _newline(1) _col(15) "95% Conf. Interval"
        di _col(5) "Coeff" _col(15) "Lower" _col(25) "Upper"
        di _col(5) %5.4f changexb`i' _col(15) %5.4f changelb`i' _col(25)
%5.4f changeub`i'
    }
    di _newline(1) in yellow "Overall across all prevalence categories"
    test _IppcXslppr_1 + _IppcXslpch_1 = _IppcXslppr_2 + _IppcXslpch_2 =
_IppcXslppr_3 + _IppcXslpch_3 = 0
    /*back transformations (some of the scalars used here were calculated
in the back-trans for the main model)*/
    if "`1'"=="logit" {
        forvalues i=1(1)3 {
            scalar up0405xb`i' = _b[_IppcXlvlch_`i']
            scalar up0405lb`i' = _b[_IppcXlvlch_`i']-
1.96*_se[_IppcXlvlch_`i']
            scalar up0405ub`i' =
_b[_IppcXlvlch_`i']+1.96*_se[_IppcXlvlch_`i']
            di _newline(2) "Back-tr Prev cat `i'" _col(25) "eff" _col(35)
"L95%" _col(45) "U95%" _col(55) "pval"
            foreach outc in up0405 up0607 change {
                /*calculate p-value*/
                scalar tempse = (`outc'ub`i' - `outc'xb`i')/1.96
                scalar tempz = `outc'xb`i'/tempse
                scalar tempp = 2*(1-normal(abs(tempz)))
                /*back transforming*/
                /*using raw predicted overall mean and difference to get
"observed" score (for eff AND CIs)*/
                scalar teff = RM`outc'+`outc'xb`i'
                scalar tl95 = RM`outc'+`outc'lb`i'
                scalar tu95 = RM`outc'+`outc'ub`i'
                /*backtrasnformations to percentages*/
                scalar bt1 = 100*1/(1+exp(-RM`outc'))
                scalar bteff = 100*1/(1+exp(-teff))
                scalar btl95 = 100*1/(1+exp(-tl95))
                scalar btu95 = 100*1/(1+exp(-tu95))
                /*and differences on back-transformations ("observed" -
predicted)*/
```

```
				scalar deff = bteff-bt1
				scalar dl95 = btl95-bt1
				scalar du95 = btu95-bt1
				if `i'==2 & "`outc'"=="up0405" {
					di "`outc'" _col(25) "%" %4.1f deff _col(35) "%" 
%4.2f dl95 _col(45) "%" %4.1f du95 _col(55) %5.3f tempp
				}
				else {
					di "`outc'" _col(25) "%" %4.1f deff _col(35) "%" 
%4.1f dl95 _col(45) "%" %4.1f du95 _col(55) %5.3f tempp
				}
			}
		}
	}

	/*prevalence and deprivation in a single variable*/
	capture drop _*
	di _newline(2)
	xi: xtmixed `indvar' i.ppd*lvlchange i.ppd*slppre i.ppd*slpchange || 
praceid: || pateid:
	/***/
	di _newline(1) in yellow "Comparing pre-qof extrapolated trend 
differences to true score differences in 04-05"
	di _newline(1) in yellow "Overall across all prevalence-deprivation 
categories"
	test _IppdXlvlch_1 = _IppdXlvlch_2 = _IppdXlvlch_3 = 0
	di _newline(1) in yellow "Comparing pre-qof extrapolated trend 
differences to true score differences in 06-07"
	forvalues i=1(1)3 {
		di _newline(1) in yellow "Prev-depr cat `i' vs 0"
		test _IppdXlvlch_`i' + 2*_IppdXslpch_`i' = 0
		scalar df = 100000
		scalar zval = sqrt(r(chi2))
		scalar up0607xb`i' = _b[_IppdXlvlch_`i'] + 2*_b[_IppdXslpch_`i']
		scalar seval = up0607xb`i'/zval
		scalar up0607lb`i' = up0607xb`i' - invttail(df,0.025)*seval
		scalar up0607ub`i' = up0607xb`i' + invttail(df,0.025)*seval
		di  _col(10) "zval = " %4.2f zval
		di _newline(1) _col(15) "95% Conf. Interval"
		di _col(5) "Coeff" _col(15) "Lower" _col(25) "Upper"
		di _col(5) %5.4f up0607xb`i' _col(15) %5.4f up0607lb`i' _col(25) 
%5.4f up0607ub`i'
	}
	di _newline(1) in yellow "Overall across all prevalence-deprivation 
categories"
	test _IppdXlvlch_1 + 2*_IppdXslpch_1 = _IppdXlvlch_2 + 
2*_IppdXslpch_2 = _IppdXlvlch_3 + 2*_IppdXslpch_3 = 0
	/***/
	di _newline(1) in yellow "Comparing 04-05 scores to 06-07 (slope)"
	forvalues i=1(1)3 {
		di _newline(1) in yellow "Prev-depr cat `i' vs 0"
		test _IppdXslppr_`i' + _IppdXslpch_`i' = 0
		scalar df = 100000
		scalar zval = sqrt(r(chi2))
		scalar changexb`i' = _b[_IppdXslppr_`i'] + _b[_IppdXslpch_`i']
		scalar seval = changexb`i'/zval
		scalar changelb`i' = changexb`i' - invttail(df,0.025)*seval
		scalar changeub`i' = changexb`i' + invttail(df,0.025)*seval
```

```
        di  _col(10) "zval = " %4.2f zval
        di _newline(1) _col(15) "95% Conf. Interval"
        di _col(5) "Coeff" _col(15) "Lower" _col(25) "Upper"
        di _col(5) %5.4f changexb`i' _col(15) %5.4f changelb`i' _col(25)
%5.4f changeub`i'
    }
    di _newline(1) in yellow "Overall across all prevalence-deprivation
categories"
    test _IppdXslppr_1 + _IppdXslpch_1 = _IppdXslppr_2 + _IppdXslpch_2 =
_IppdXslppr_3 + _IppdXslpch_3 = 0
    /*back transformations (some of the scalars used here were calculated
in the back-trans for the main model)*/
    if "`1'"=="logit" {
        forvalues i=1(1)3 {
            scalar up0405xb`i' = _b[_IppdXlvlch_`i']
            scalar up0405lb`i' = _b[_IppdXlvlch_`i']-
1.96*_se[_IppdXlvlch_`i']
            scalar up0405ub`i' =
_b[_IppdXlvlch_`i']+1.96*_se[_IppdXlvlch_`i']
            di _newline(2) "Back-tr Prev-depr cat `i'" _col(25) "eff"
_col(35) "L95%" _col(45) "U95%" _col(55) "pval"
            foreach outc in up0405 up0607 change {
                /*calculate p-value*/
                scalar tempse = (`outc'ub`i' - `outc'xb`i')/1.96
                scalar tempz = `outc'xb`i'/tempse
                scalar tempp = 2*(1-normal(abs(tempz)))
                /*back transforming*/
                /*using raw predicted overall mean and difference to get
"observed" score (for eff AND CIs)*/
                scalar teff = RM`outc'+`outc'xb`i'
                scalar tl95 = RM`outc'+`outc'lb`i'
                scalar tu95 = RM`outc'+`outc'ub`i'
                /*backtrasnformations to percentages*/
                scalar bt1 = 100*1/(1+exp(-RM`outc'))
                scalar bteff = 100*1/(1+exp(-teff))
                scalar btl95 = 100*1/(1+exp(-tl95))
                scalar btu95 = 100*1/(1+exp(-tu95))
                /*and differences on back-transformations ("observed" -
predicted)*/
                scalar deff = bteff-bt1
                scalar dl95 = btl95-bt1
                scalar du95 = btu95-bt1
                di "`outc'" _col(25) "%" %4.1f deff _col(35) "%" %4.1f
dl95 _col(45) "%" %4.1f du95 _col(55) %5.3f tempp
            }
        }
    }

    /*close log*/
    qui log close log1
end
```
