# Supplementary material for: Regression based quasi-experimental approach when randomisation is not an option: interrupted time series analysis
Source: BMJ. 2015 Jun 9;350:h2750. doi: 10.1136/bmj.h2750 (PMC4460815; doi:10.1136/bmj.h2750)
Supplement: Supplementary file 3 [file kone024026.ww3_default.pdf]

Web appendix 3: Code file for expert ITS analysis, approach A

```
/*final analyses - linear*/
version 10.1
cd "S:\QOF_GPRD\GPRD\Analysis\David\"
use "S:\QOF_GPRD\GPRD\STATA\code\final_dataset\finalwide.dta"
qui drop *1999 indscorelogit*
/*drop A-I indicators*/
qui drop if indgroup3==12
/*set to missing data from 2 practices for which 06/07 data is not
complete - across all time points*/
qui drop if praceid==164020 | praceid==609533
tempfile temp

set more off
set linesize 90
set maxiter 50 /*maximum number of iterations to take care of non-
convergence*/

sort studycell3
by studycell3: tab indname
sort indtype
by indtype: tab indname

qui {
    capture drop premean preslope postchange uplift0405 uplift0607
predict0405 predict0607
    gen premean=(indscore2000+indscore2001+indscore2002)/3
    gen preslope =(indscore2002 - indscore2000)/2
    gen postchange = indscore2006-indscore2004
    gen predict0405 = (11*indscore2002+2*indscore2001-7*indscore2000)/6
    replace predict0405=1 if predict0405>1 & predict0405!=.
    replace predict0405=0 if predict0405<0 & predict0405!=.
    gen predict0607 = (17* indscore2002+2* indscore2001-13*
indscore2000)/6
    replace predict0607=1 if predict0607>1 & predict0607!=.
    replace predict0607=0 if predict0607<0 & predict0607!=.
    gen uplift0607= indscore2006-predict0607
    gen uplift0405 = indscore2004-predict0405
    /*control for denominators*/
    capture drop denpostchange denuplift0405 denuplift0607
    gen denpostchange = den2006-den2004
    gen denuplift0607= den2006-(17* den2002+2* den2001-13* den2000)/6
    gen denuplift0405 = den2004-(11*den2002+2*den2001-7*den2000)/6
    /*control for sex*/
    capture drop sexpostchange sexuplift0405 sexuplift0607
    gen sexpostchange = sex2006-sex2004
    gen sexuplift0607= sex2006-(17* sex2002+2* sex2001-13* sex2000)/6
    gen sexuplift0405 = sex2004-(11*sex2002+2*sex2001-7*sex2000)/6
    /*control for age*/
    capture drop agepostchange ageuplift0405 ageuplift0607
    gen agepostchange = age2006-age2004
    gen ageuplift0607= age2006-(17* age2002+2* age2001-13* age2000)/6
    gen ageuplift0405 = age2004-(11*age2002+2*age2001-7*age2000)/6
}
sort indnum
qui save temp1.dta, replace
```

```
/*collapse and merge to create residuals - in a different do file since
it's repeated a lot in this piece of code*/
do collapse_and_merge_linear
/*generate the three variables to be used in analyses*/
qui gen adjuplift0405=.
foreach x in ASTHMA4 BP2 BP4 CHD3 CHD5 CHD7 COPD4 DM11 DM14 DM16 DM5 MH3
MH4 STROKE3 STROKE5 STROKE7 THYROID2 /*
*/ CHD10 CHD12 COPD8 DM18 LVD3 STROKE10 B10 B3 B6 B8 B9 C3 C5 C6 C7 B2i
B2ii C2(rev) C4(rev) /*
*/ D11 D5 D6(rev) D7(rev) D8(rev) D9(rev) {
    *di "`x'" _continue
    qui {
        regress uplift0405 predict0405RES denuplift0405 ageuplift0405
sexuplift0405 if indname=="`x'"
        adjust predict0405RES denuplift0405=0 ageuplift0405=0
sexuplift0405=0 if e(sample), ci generate(adjmean)
        predict resd if e(sample), residuals
        replace adjuplift0405 = adjmean + resd if indname=="`x'"
        drop adjmean res
    }
}
qui gen adjuplift0607=.
foreach x in ASTHMA4 BP2 BP4 CHD3 CHD5 CHD7 COPD4 DM11 DM14 DM16 DM5 MH3
MH4 STROKE3 STROKE5 STROKE7 THYROID2 /*
*/ CHD10 CHD12 COPD8 DM18 LVD3 STROKE10 B10 B3 B6 B8 B9 C3 C5 C6 C7 B2i
B2ii C2(rev) C4(rev) /*
*/ D11 D5 D6(rev) D7(rev) D8(rev) D9(rev) {
    *di "`x'" _continue
    qui {
        regress  uplift0607 predict0607RES denuplift0607 ageuplift0607
sexuplift0607 if indname=="`x'"
        adjust predict0607RES denuplift0607=0 ageuplift0607=0
sexuplift0607=0 if e(sample), ci generate(adjmean)
        predict resd if e(sample), residuals
        replace adjuplift0607 = adjmean + resd if indname=="`x'"
        drop adjmean res
    }
}
qui gen adjpostchange=.
foreach x in ASTHMA4 BP2 BP4 CHD3 CHD5 CHD7 COPD4 DM11 DM14 DM16 DM5 MH3
MH4 STROKE3 STROKE5 STROKE7 THYROID2 /*
*/ CHD10 CHD12 COPD8 DM18 LVD3 STROKE10 B10 B3 B6 B8 B9 C3 C5 C6 C7 B2i
B2ii C2(rev) C4(rev) /*
*/ D11 D5 D6(rev) D7(rev) D8(rev) D9(rev) {
    *di "`x'" _continue
    qui {
        regress  postchange indscore2004RES denpostchange agepostchange
sexpostchange if indname=="`x'"
        adjust indscore2004RES denpostchange=0 agepostchange=0
sexpostchange=0 if e(sample), ci generate(adjmean)
        predict resd if e(sample), residuals
        replace adjpostchange = adjmean+ resd if indname=="`x'"
        drop adjmean res
    }
}
/*save temp file*/
qui save `temp', replace
```

```
/*log output*/
*qui log using linearlog, name(log1) text replace
qui log using linearlog, name(log2) smcl replace

/*UPLIFT0405*/
/*Uplift0405 FIXED INDICATORS - Controlling for RTTM and denominators,
age, gender*/
xi, noomit: xtmixed adjuplift0405 i.indnum, noconstant || /*
*/ _all: R.praceid || indnum: , ml emonly
adjust if e(sample), by(indgroup3) ci replace
/*overall comparisons and newmal_keuls*/
do pair_comparison_14Oct10
/*Uplift0405 FIXED indicators - controlling for RTTM, predict0405 and
denominators, age, gender*/
qui use `temp',clear
qui xi, noomit: xtmixed adjuplift0405 premeanIM preslopeIM den2004IM
age2004IM sex2004IM i.indnum, noconstant || /*
*/_all: R.praceid || indnum: , ml emonly emiterate(1)
qui keep if e(sample)==1
qui drop *IM *RES
qui save temp1.dta, replace
do collapse_and_merge_linear
xi, noomit: xtmixed adjuplift0405 premeanIM preslopeIM den2004IM
age2004IM sex2004IM i.indnum, noconstant || /*
*/_all: R.praceid || indnum: , ml emonly
capture scalar t1 = _b[preslopeIM]
if _rc==0 {
    local strtmp = "premeanIM preslopeIM"
}
else {
    local strtmp = "premeanIM"
}
adjust `strtmp' den2004IM age2004IM sex2004IM if e(sample), by(indgroup3)
ci replace
/*overall comparisons and newmal_keuls*/
do pair_comparison_14Oct10

/*UPLIFT0607*/
/*Uplift0607 FIXED INDICATORS - Controlling for RTTM and denominators,
age, gender*/
qui use `temp',clear
xi, noomit: xtmixed adjuplift0607 i.indnum, noconstant || /*
*/ _all: R.praceid || indnum: , ml emonly
adjust if e(sample), by(indgroup3) ci replace
/*overall comparisons and newmal_keuls*/
do pair_comparison_14Oct10
/*Uplift0607 FIXED INDICATORS - controlling for RTTM, predict0405 and
denominators, age, gender*/
qui use `temp',clear
qui xi, noomit: xtmixed adjuplift0607 premeanIM preslopeIM den2006IM
age2006IM sex2006IM i.indnum, noconstant || /*
*/ _all: R.praceid || indnum: , ml emonly emiterate(1)
qui keep if e(sample)==1
qui drop *IM *RES
qui save temp1.dta, replace
do collapse_and_merge_linear
```

```
xi, noomit: xtmixed adjuplift0607 premeanIM preslopeIM den2006IM
age2006IM sex2006IM i.indnum, noconstant || /*
*/ _all: R.praceid || indnum: , ml emonly
capture scalar t1 = _b[preslopeIM]
if _rc==0 {
    local strtmp = "premeanIM preslopeIM"
}
else {
    local strtmp = "premeanIM"
}
adjust `strtmp' den2006IM age2006IM sex2006IM if e(sample), by(indgroup3)
ci replace
/*overall comparisons and newmal_keuls*/
do pair_comparison_14Oct10

/*CHANGE 0405 TO 0607*/
/*Change0405 to 0607 FIXED INDICATORS - Controlling for RTTM and
denominators, age, gender*/
qui use `temp',clear
xi, noomit: xtmixed adjpostchange i.indnum, noconstant || /*
*/ _all: R.praceid || indnum: , ml emonly
adjust if e(sample), by(indgroup3) ci replace
/*overall comparisons and newmal_keuls*/
do pair_comparison_14Oct10
/*Change0405 to 0607 FIXED indicators - Controlling for RTTM, score in
0405, den, age and sex*/
qui use `temp',clear
qui xi, noomit: xtmixed adjpostchange indscore2004IM den2006IM age2006IM
sex2006IM i.indnum, noconstant ||
*/ _all: R.praceid || indnum: , ml emonly emiterate(1)
qui keep if e(sample)==1
qui drop *IM *RES
qui save temp1.dta, replace
do collapse_and_merge_linear
xi, noomit: xtmixed adjpostchange indscore2004IM den2006IM age2006IM
sex2006IM i.indnum, noconstant ||
*/ _all: R.praceid || indnum: , ml emonly
adjust indscore2004IM den2006IM age2006IM sex2006IM if e(sample),
by(indgroup3) ci replace
/*overall comparisons and newmal_keuls*/
do pair_comparison_14Oct10

capture erase temp1.dta
/*For each individual indicator run the below commands:*/
forvalues i=1(1)65 {
    qui use `temp',clear
    qui keep if indnum==`i'
    qui count
    if r(N)>0 {
        di _newline(1) indname[1]
        regress  uplift0405 predict0405 denuplift0405 ageuplift0405
sexuplift0405
        adjust predict0405 denuplift0405=0 ageuplift0405=0
sexuplift0405=0 if e(sample), ci

        regress  uplift0607 predict0607 denuplift0607 ageuplift0607
sexuplift0607
```

```
        adjust predict0607 denuplift0607=0 ageuplift0607=0
sexuplift0607=0 if e(sample), ci

        regress  postchange indscore2004 denpostchange agepostchange
sexpostchange
        adjust indscore2004 denpostchange=0 agepostchange=0
sexpostchange=0 if e(sample), ci
    }
}

/*close log*/
*qui log close log1
qui log close log2
```
