# Supplementary material for: Regression based quasi-experimental approach when randomisation is not an option: interrupted time series analysis
Source: BMJ. 2015 Jun 9;350:h2750. doi: 10.1136/bmj.h2750 (PMC4460815; doi:10.1136/bmj.h2750)
Supplement: Supplementary file 4 [file kone024026.ww4_default.pdf]

Web appendix 4: Code file for expert ITS analysis, approach B

```
/*  1: untrasformed scores
    2: practices contributing fully within each year
    3: year as continuous for last 3 available years (or 2 if only 2
available), including interactions
    4: within condition controls*/
program reganal
    local dir1 = "P:\Evan\GPRD\Indicator removal\logs\"
    local dir2 = "P:\Evan\GPRD\Indicator removal\"
    local dir3 = "P:\Evan\GPRD\Indicator removal\matrices\"
    capture mkdir "`dir1'"
    capture mkdir "`dir3'"
    cd "`dir1'"
    tempfile temp1 temp2
      tempvar tsum txb tstdp tD se_fix se_u1 se_u2 tempden
      qui ssc install metaan
    set more off
      *set trace on
      *set tracedepth 1

    /*main analysis*/
    /*notes:
        1)if indcode used as fixed cannot use any indicator
categorisation variables and hence need to run separate analyses for each
domain
        2) run regression only on active indicators and use model to
predict and then compare with observed
           3) in terms of the predictions there are two options
                 a) use the margins command to estimate an overall effect
and CI and compare that to the observed - needs to be adjusted for each
time point
                 b) use the predict command to calculate the predictions
and their SE, calculate an overall effect (MA) and compare - either way
we observe
                       a healthy increase in SE as the years increase*/
    /*log open*/
    qui log using "4a-analysis_linear_main", name(log1) smcl replace
      /*analysis 1 - influenza immunisation*/
    qui use "`dir2'dataset_inflimm.dta", clear
    qui keep if year>3 & prctgrp1==1
    qui keep if inlist(indcode,8,26,40,61,92,6)
    di _newline(3) "Influenza immunisation model"
    /*model*/
    xtmixed indscore i.indcode year i.indcode#c.year prev listsize
i.depquint i.region if indstatus==0 || pracid: c.year
      /*predict*/
      qui predict `txb', fitted
      qui replace `txb'=0 if `txb'<0
      qui replace `txb'=100 if `txb'>100
      qui predict `se_fix',  stdp
      qui predict `se_u1' `se_u2', reses
      qui gen `tstdp'=  sqrt(`se_fix'^2 + `se_u1'^2 + `se_u2'^2)
      qui drop `se_fix' `se_u1' `se_u2'
      /*add the variance of the observed - July 2013*/
      qui gen `tempden'=max(1,den)
```

```
      qui replace `tstdp'=sqrt(`tstdp'^2 + indscore/100*(1-
indscore/100)/`tempden')
      /*now use meta-analysis methods to calculate between-observation
variation and calculate overall variance taking between and within into
account*/
      qui generate `tD' = indscore-`txb'
      qui replace `tstdp'=. if `tD'==.
      local str1 = "Asthma7"
      matrix pred`str1'=J(8,8,.)
      forvalues i=1(1)8 {
            qui metaan `tD' `tstdp' if indcode==8 & year==`=`i'+3', bdl
reps(1000)
            /*use the meta-analysis results, everything is there*/
            scalar tD=r(eff)
         scalar tVAR = r(effvar)
            scalar tN=r(df)+1
            scalar tLD=r(efflo)
            scalar tUD=r(effup)
            scalar tSE=abs((tUD-tLD)/(2*1.96))
            scalar pval=exp(-0.717*abs(tD)/tSE - 0.416*((abs(tD)/tSE)^2))
            /*alternatively use a paired t-test*/
            /*qui sum `tD' if indcode==8 & year==`=`i'+3'
            scalar tD=r(mean)
            scalar tN=r(N)
            scalar tSE=sqrt(tVAR)/sqrt(tN)
            scalar tSTAT=tD/tSE
            scalar tP=ttail(tN-1,abs(tSTAT))*2*/
            /*add data to matrix*/
            qui sum indscore if indcode==8 & year==`=`i'+3' & `tD'!=.
      /*making sure have exactly the same sample in observed and
predicted, just in case*/
            matrix pred`str1'[1,`i']=r(mean)
            qui sum `txb' if indcode==8 & year==`=`i'+3' & `tD'!=.
      /*making sure have exactly the same sample in observed and
predicted, just in case*/
            matrix pred`str1'[2,`i']=r(mean)
            matrix pred`str1'[3,`i']=tD
            matrix pred`str1'[4,`i']=tN
            matrix pred`str1'[5,`i']=tVAR
            matrix pred`str1'[6,`i']=tSE
            matrix pred`str1'[7,`i']=tLD
            matrix pred`str1'[8,`i']=tUD
      }
      /*reset matrix rownames*/
      mat rownames pred`str1' = obs pred d N var se l95d u95d
      mat colnames pred`str1' = y1 y2 y3 y4 y5 y6 y7 y8
      di _newline(1) "Predictions for `str1' over time"
      matrix list pred`str1'
      qui drop `txb' `tstdp' `tD' `tempden'

      /*analysis 2 - bloodpressure*/
    qui use "`dir2'dataset_bloodpress.dta", clear
    qui keep if year>3 & prctgrp1==1
    di _newline(3) "Blood pressure models"
    /*indicator sets*/
    local indset19 = "inlist(indcode,19,11,54,87,16)"
    local indset54 = "inlist(indcode,19,11,54,87,45,57)"
    local indset87 = "inlist(indcode,19,11,54,87,89)"
```

```
      local str19="CHD5"
      local str54="DM11"
      local str87="Stroke5"
      matrix pred`str19'=J(8,8,.)
      matrix pred`str54'=J(8,8,.)
      matrix pred`str87'=J(8,8,.)
    /*loop*/
    foreach x in 19 54 87 {
            /*model*/
        xtmixed indscore i.indcode year i.indcode#c.year prev listsize
i.depquint i.region if indstatus==0 & year>7 & `indset`x'' || pracid:
c.year
      /*predict*/
            qui predict `txb', fitted
            qui replace `txb'=0 if `txb'<0
            qui replace `txb'=100 if `txb'>100
            qui predict `se_fix',  stdp
            qui predict `se_u1' `se_u2', reses
            qui gen `tstdp'=  sqrt(`se_fix'^2 + `se_u1'^2 + `se_u2'^2)

            qui drop `se_fix' `se_u1' `se_u2'
      /*add the variance of the observed - July 2013*/
          qui gen `tempden'=max(1,den)
          qui replace `tstdp'=sqrt(`tstdp'^2 + indscore/100*(1-
indscore/100)/`tempden')
      /*now use meta-analysis methods to calculate between-observation
variation and calculate overall variance taking between and within into
account*/
      qui generate `tD' = indscore-`txb'
      qui replace `tstdp'=. if `tD'==.
            /*meta-analysis loop for each year*/
            forvalues i=1(1)8 {
                  qui metaan `tD' `tstdp' if indcode==`x' &
year==`=`i'+3', bdl reps(1000)
                  /*use the meta-analysis results, everything is there*/
                  scalar tD=r(eff)
                  scalar tVAR = r(effvar)
                  scalar tN=r(df)+1
            scalar tLD=r(efflo)
            scalar tUD=r(effup)
            scalar tSE=abs((tUD-tLD)/(2*1.96))
            scalar pval=exp(-0.717*abs(tD)/tSE - 0.416*((abs(tD)/tSE)^2))
                  /*add data to matrix*/
                  qui sum indscore if indcode==`x' & year==`=`i'+3' &
`tD'!=.          /*making sure have exactly the same sample in observed
and predicted, just in case*/
                  matrix pred`str`x''[1,`i']=r(mean)
                  qui sum `txb' if indcode==`x' & year==`=`i'+3' & `tD'!=.
             /*making sure have exactly the same sample in observed and
predicted, just in case*/
                  matrix pred`str`x''[2,`i']=r(mean)
                  matrix pred`str`x''[3,`i']=tD
                  matrix pred`str`x''[4,`i']=tN
                  matrix pred`str`x''[5,`i']=tVAR
                  matrix pred`str`x''[6,`i']=tSE
                  matrix pred`str`x''[7,`i']=tLD

                  matrix pred`str`x''[8,`i']=tUD
```

```
            }
            /*reset matrix rownames*/
            mat rownames pred`str`x'' = obs pred d N var se l95d u95d
            mat colnames pred`str`x'' = y1 y2 y3 y4 y5 y6 y7 y8
            di _newline(1) "Predictions for `str`x'' over time"
            matrix list pred`str`x''
      qui drop `txb' `tstdp' `tD' `tempden'
      }

      /*analysis 3 - cholesterol*/
    qui use "`dir2'dataset_cholesterol.dta", clear
    qui keep if year>3 & prctgrp1==1
    di _newline(3) "Cholesterol models"
    /*indicator sets*/
    local indset21 = "inlist(indcode,21,59,89,16)"
    local indset59 = "inlist(indcode,21,59,89,45,57)"
      local str21="CHD7"
      local str59="DM16"
      matrix pred`str21'=J(8,8,.)
      matrix pred`str59'=J(8,8,.)
    /*loop*/
    foreach x in 21 59 {
            /*model*/
        /*xtmixed indscore i.indcode year i.indcode#c.year prev listsize
i.depquint i.region || pracid: c.year indcode not converging*/
        xtmixed indscore i.indcode year i.indcode#c.year prev listsize
i.depquint i.region if indstatus==0 & year>7 & `indset`x'' || pracid:
c.year
      /*predict*/
            qui predict `txb', fitted
            qui replace `txb'=0 if `txb'<0
            qui replace `txb'=100 if `txb'>100
            qui predict `se_fix',  stdp
            qui predict `se_u1' `se_u2', reses
            qui gen `tstdp'=  sqrt(`se_fix'^2 + `se_u1'^2 + `se_u2'^2)

            qui drop `se_fix' `se_u1' `se_u2'
      /*add the variance of the observed - July 2013*/
          qui gen `tempden'=max(1,den)
          qui replace `tstdp'=sqrt(`tstdp'^2 + indscore/100*(1-
indscore/100)/`tempden')
      /*now use meta-analysis methods to calculate between-observation
variation and calculate overall variance taking between and within into
account*/
      qui generate `tD' = indscore-`txb'
      qui replace `tstdp'=. if `tD'==.
            /*meta-analysis loop for each year*/
            forvalues i=1(1)8 {
                  qui metaan `tD' `tstdp' if indcode==`x' &
year==`=`i'+3', bdl reps(1000)
                  /*use the meta-analysis results, everything is there*/
                  scalar tD=r(eff)
                  scalar tVAR = r(effvar)
                  scalar tN=r(df)+1
            scalar tLD=r(efflo)
            scalar tUD=r(effup)
            scalar tSE=abs((tUD-tLD)/(2*1.96))
            scalar pval=exp(-0.717*abs(tD)/tSE - 0.416*((abs(tD)/tSE)^2))
```

```
                /*add data to matrix*/
                qui sum indscore if indcode==`x' & year==`=`i'+3' &
`tD'!=.    /*making sure have exactly the same sample in observed and
predicted, just in case*/
                matrix pred`str`x''[1,`i']=r(mean)
                qui sum `txb' if indcode==`x' & year==`=`i'+3' & `tD'!=.
           /*making sure have exactly the same sample in observed and
predicted, just in case*/
                matrix pred`str`x''[2,`i']=r(mean)
                matrix pred`str`x''[3,`i']=tD
                matrix pred`str`x''[4,`i']=tN
                matrix pred`str`x''[5,`i']=tVAR
                matrix pred`str`x''[6,`i']=tSE
                matrix pred`str`x''[7,`i']=tLD

                matrix pred`str`x''[8,`i']=tUD
           }
           /*reset matrix rownames*/
           mat rownames pred`str`x'' = obs pred d N var se l95d u95d
           mat colnames pred`str`x'' = y1 y2 y3 y4 y5 y6 y7 y8
           di _newline(1) "Predictions for `str`x'' over time"
           matrix list pred`str`x''
      qui drop `txb' `tstdp' `tD' `tempden'
      }

      /*analysis 4 - glucose*/
    qui use "`dir2'dataset_glucose.dta", clear
    qui keep if year>3 & prctgrp1==1
    qui keep if inlist(indcode,48,45,57)
    di _newline(3) "Glucose model"
    /*model*/
    xtmixed indscore i.indcode year i.indcode#c.year prev listsize
i.depquint i.region if indstatus==0 & year>7 || pracid: c.year
      /*predict*/
      qui predict `txb', fitted
      qui replace `txb'=0 if `txb'<0
      qui replace `txb'=100 if `txb'>100
      qui predict `se_fix',  stdp
      qui predict `se_u1' `se_u2', reses
      qui gen `tstdp'=  sqrt(`se_fix'^2 + `se_u1'^2 + `se_u2'^2)
      qui drop `se_fix' `se_u1' `se_u2'
      /*add the variance of the observed - July 2013*/
      qui gen `tempden'=max(1,den)
      qui replace `tstdp'=sqrt(`tstdp'^2 + indscore/100*(1-
indscore/100)/`tempden')
      /*now use meta-analysis methods to calculate between-observation
variation and calculate overall variance taking between and within into
account*/
      qui generate `tD' = indscore-`txb'
      qui replace `tstdp'=. if `tD'==.
      /*margins*/
      local str1 = "DM5"
      matrix pred`str1'=J(8,8,.)
      forvalues i=1(1)8 {
           qui metaan `tD' `tstdp' if indcode==48 & year==`=`i'+3', bdl
reps(1000)
           /*use the meta-analysis results, everything is there*/
           scalar tD=r(eff)
```

```
        scalar tVAR = r(effvar)
            scalar tN=r(df)+1
            scalar tLD=r(efflo)
            scalar tUD=r(effup)
            scalar tSE=abs((tUD-tLD)/(2*1.96))
            scalar pval=exp(-0.717*abs(tD)/tSE - 0.416*((abs(tD)/tSE)^2))
            /*add data to matrix*/
            qui sum indscore if indcode==48 & year==`=`i'+3' & `tD'!=.
      /*making sure have exactly the same sample in observed and
predicted, just in case*/
            matrix pred`str1'[1,`i']=r(mean)
            qui sum `txb' if indcode==48 & year==`=`i'+3' & `tD'!=.
      /*making sure have exactly the same sample in observed and
predicted, just in case*/
            matrix pred`str1'[2,`i']=r(mean)
            matrix pred`str1'[3,`i']=tD
            matrix pred`str1'[4,`i']=tN
            matrix pred`str1'[5,`i']=tVAR
            matrix pred`str1'[6,`i']=tSE
            matrix pred`str1'[7,`i']=tLD
            matrix pred`str1'[8,`i']=tUD
      }
      /*reset matrix rownames*/
      mat rownames pred`str1' = obs pred d N var se l95d u95d
      mat colnames pred`str1' = y1 y2 y3 y4 y5 y6 y7 y8
      di _newline(1) "Predictions for `str1' over time"
      matrix list pred`str1'
      qui drop `txb' `tstdp' `tD' `tempden'

      /*analysis 5 - lithium*/
    qui use "`dir2'dataset_lithium.dta", clear
    qui keep if year>3 & prctgrp1==1
    qui keep if inlist(indcode,75,76)
    di _newline(3) "Lithium model"
    /*model*/
    /*xtmixed indscore i.indcode year i.indcode#c.year prev listsize
i.depquint i.region || pracid: c.year indcode not converging*/
    xtmixed indscore i.indcode year i.indcode#c.year prev listsize
i.depquint i.region if indstatus==0 || pracid: c.year
      /*predict*/
      qui predict `txb', fitted
      qui replace `txb'=0 if `txb'<0
      qui replace `txb'=100 if `txb'>100
      qui predict `se_fix',  stdp
      qui predict `se_u1' `se_u2', reses
      qui gen `tstdp'=  sqrt(`se_fix'^2 + `se_u1'^2 + `se_u2'^2)
      qui drop `se_fix' `se_u1' `se_u2'
      /*add the variance of the observed - July 2013*/
      qui gen `tempden'=max(1,den)
      qui replace `tstdp'=sqrt(`tstdp'^2 + indscore/100*(1-
indscore/100)/`tempden')
      /*now use meta-analysis methods to calculate between-observation
variation and calculate overall variance taking between and within into
account*/
      qui generate `tD' = indscore-`txb'
      qui replace `tstdp'=. if `tD'==.
      /*margins*/
      local str1 = "MH3"
```

```
      matrix pred`str1'=J(8,8,.)
      forvalues i=1(1)8 {
            qui metaan `tD' `tstdp' if indcode==75 & year==`=`i'+3', bdl
reps(1000)
            /*use the meta-analysis results, everything is there*/
            scalar tD=r(eff)
         scalar tVAR = r(effvar)
            scalar tN=r(df)+1
            scalar tLD=r(efflo)
            scalar tUD=r(effup)
            scalar tSE=abs((tUD-tLD)/(2*1.96))
            scalar pval=exp(-0.717*abs(tD)/tSE - 0.416*((abs(tD)/tSE)^2))
            /*add data to matrix*/
            qui sum indscore if indcode==75 & year==`=`i'+3' & `tD'!=.
      /*making sure have exactly the same sample in observed and
predicted, just in case*/
            matrix pred`str1'[1,`i']=r(mean)
            qui sum `txb' if indcode==75 & year==`=`i'+3' & `tD'!=.
      /*making sure have exactly the same sample in observed and
predicted, just in case*/
            matrix pred`str1'[2,`i']=r(mean)
            matrix pred`str1'[3,`i']=tD
            matrix pred`str1'[4,`i']=tN
            matrix pred`str1'[5,`i']=tVAR
            matrix pred`str1'[6,`i']=tSE
            matrix pred`str1'[7,`i']=tLD
            matrix pred`str1'[8,`i']=tUD
      }
      /*reset matrix rownames*/
      mat rownames pred`str1' = obs pred d N var se l95d u95d
      mat colnames pred`str1' = y1 y2 y3 y4 y5 y6 y7 y8
      di _newline(1) "Predictions for `str1' over time"
      matrix list pred`str1'
      qui drop `txb' `tstdp' `tD' `tempden'

      /*analysis 6 - medication review*/
    qui use "`dir2'dataset_medreview.dta", clear
    qui keep if year>3 & prctgrp1==1
    di _newline(3) "Medication review model"
    /*model*/
    /*xtmixed indscore i.indcode year i.indcode#c.year prev listsize
i.depquint i.region || pracid: c.year indcode not converging*/
    xtmixed indscore i.indcode year i.indcode#c.year prev listsize
i.depquint i.region if indstatus==0 & year>7 || pracid: c.year
      /*predict*/
      qui predict `txb', fitted
      qui replace `txb'=0 if `txb'<0
      qui replace `txb'=100 if `txb'>100
      qui predict `se_fix',  stdp
      qui predict `se_u1' `se_u2', reses
      qui gen `tstdp'=  sqrt(`se_fix'^2 + `se_u1'^2 + `se_u2'^2)
      qui drop `se_fix' `se_u1' `se_u2'
      /*add the variance of the observed - July 2013*/
      qui gen `tempden'=max(1,den)
      qui replace `tstdp'=sqrt(`tstdp'^2 + indscore/100*(1-
indscore/100)/`tempden')
```

```
      /*now use meta-analysis methods to calculate between-observation
variation and calculate overall variance taking between and within into
account*/
      qui generate `tD' = indscore-`txb'
      qui replace `tstdp'=. if `tD'==.
      /*margins*/
      local str1 = "EPI7"
      matrix pred`str1'=J(8,8,.)
      forvalues i=1(1)8 {
            qui metaan `tD' `tstdp' if indcode==65 & year==`=`i'+3', bdl
reps(1000)
            /*use the meta-analysis results, everything is there*/
            scalar tD=r(eff)
        scalar tVAR = r(effvar)
            scalar tN=r(df)+1
            scalar tLD=r(efflo)
            scalar tUD=r(effup)
            scalar tSE=abs((tUD-tLD)/(2*1.96))
            scalar pval=exp(-0.717*abs(tD)/tSE - 0.416*((abs(tD)/tSE)^2))
            /*add data to matrix*/
            qui sum indscore if indcode==65 & year==`=`i'+3' & `tD'!=.
      /*making sure have exactly the same sample in observed and
predicted, just in case*/
            matrix pred`str1'[1,`i']=r(mean)
            qui sum `txb' if indcode==65 & year==`=`i'+3' & `tD'!=.
      /*making sure have exactly the same sample in observed and
predicted, just in case*/
            matrix pred`str1'[2,`i']=r(mean)
            matrix pred`str1'[3,`i']=tD
            matrix pred`str1'[4,`i']=tN
            matrix pred`str1'[5,`i']=tVAR
            matrix pred`str1'[6,`i']=tSE
            matrix pred`str1'[7,`i']=tLD
            matrix pred`str1'[8,`i']=tUD
      }
      /*reset matrix rownames*/
      mat rownames pred`str1' = obs pred d N var se l95d u95d
      mat colnames pred`str1' = y1 y2 y3 y4 y5 y6 y7 y8
      di _newline(1) "Predictions for `str1' over time"
      matrix list pred`str1'
      qui drop `txb' `tstdp' `tD' `tempden'

    /*save matrices to use in further analyses*/
    foreach x in Asthma7 CHD5 DM11 Stroke5 CHD7 DM16 DM5 MH3 EPI7 {
        qui clear
        qui svmat pred`x'
        qui compress
        qui save "`dir3'4a-matrix`x'.dta", replace
    }

    *set trace on
    *set tracedepth 1
    set linesize 180
    /*generate results table with t-tests*/
    local cl1 = 10
    forvalues i=2(1)8 {
        local cl`i' = `cl`=`i'-1'' + 21
    }
```

```
      di _newline(2) in green "Table with observed(predicted) scores / # of practices (overall variance) / meta-analysed difference (95% CI)"
    di in green _col(`cl1') "04/05" _col(`cl2') "05/06" _col(`cl3') "06/07" _col(`cl4') "07/08" _col(`cl5') "08/09" _col(`cl6') "09/10" _col(`cl7') "10/11" _col(`cl8') "11/12"
    foreach x in Asthma7 CHD5 DM11 Stroke5 CHD7 DM16 DM5 MH3 EPI7 {
        /*get data from matrices*/
        forvalues i=1(1)8 {
            scalar m1`i' = pred`x'[1,`i']
            scalar m2`i' = pred`x'[2,`i']
            scalar N`i' = pred`x'[4,`i']
            scalar var`i' = pred`x'[5,`i']
            scalar df`i'=pred`x'[3,`i']
            scalar dfl`i'=pred`x'[7,`i']
            scalar dfu`i'=pred`x'[8,`i']
        }
        di in green "`x'" in yellow _col(`cl1') %3.1f m11 "(" %3.1f m21 ")" _col(`cl2') %3.1f m12 "(" %3.1f m22 ")" _col(`cl3') %3.1f m13 "(" %3.1f m23 ")" _col(`cl4') %3.1f m14 "(" %3.1f m24 ")" /*
        */ _col(`cl5') %3.1f m15 "(" %3.1f m25 ")" _col(`cl6') %3.1f m16 "(" %3.1f m26 ")" _col(`cl7') %3.1f m17 "(" %3.1f m27 ")" _col(`cl8') %3.1f m18 "(" %3.1f m28 ")"
        di in green "  N(var)" in yellow _col(`cl1') %3.0f N1 "(" %3.2f var1 ")" _col(`cl2') %3.0f N2 "(" %3.2f var2 ")" _col(`cl3') %3.0f N3 "(" %3.2f var3 ")" _col(`cl4') %3.0f N4 "(" %3.2f var4 ")" /*
        */ _col(`cl5') %3.0f N5 "(" %3.2f var5 ")" _col(`cl6') %3.0f N6 "(" %3.2f var6 ")" _col(`cl7') %3.0f N7 "(" %3.2f var7 ")" _col(`cl8') %3.0f N8 "(" %3.2f var8 ")"
        di in green "  D(CI)" in yellow _col(`cl1') %3.2f df1 "(" %3.2f dfl1 "," %3.2f dfu1 ")" _col(`cl2') %3.2f df2 "(" %3.2f dfl2 "," %3.2f dfu2 ")" _col(`cl3') %3.2f df3 "(" %3.2f dfl3 "," %3.2f dfu3 ")" /*
        */ _col(`cl4') %3.2f df4 "(" %3.2f dfl4 "," %3.2f dfu4 ")" _col(`cl5') %3.2f df5 "(" %3.2f dfl5 "," %3.2f dfu5 ")" _col(`cl6') %3.2f df6 "(" %3.2f dfl6 "," %3.2f dfu6 ")" /*
        */ _col(`cl7') %3.2f df7 "(" %3.2f dfl7 "," %3.2f dfu7 ")" _col(`cl8') %3.2f df8 "(" %3.2f dfl8 "," %3.2f dfu8 ")"
    }
    *di in green "N" in yellow _col(`cl1') %4.0f N1 _col(`cl2') %4.0f N2 _col(`cl3') %4.0f N3 _col(`cl4') %4.0f N4 _col(`cl5') %4.0f N5 _col(`cl6') %4.0f N6 _col(`cl7') %4.0f N7 _col(`cl8') %4.0f N8

      /*log close*/
    qui log close log1
end
```
